# Diffusion mechanism and adsorbed-phase classification—molecular simulation insights from Lennard-Jones fluid on MOFs

**DOI:** 10.1016/j.isci.2025.112181

**Published:** 2025-03-08

**Authors:** Haonan Chen, Sagar Saren, Xuetao Liu, Ji Hwan Jeong, Takahiko Miyazaki, Young-Deuk Kim, Kyaw Thu

**Affiliations:** 1 Department of Advanced Environmental Science and Engineering, Faculty of Engineering Sciences, Kyushu University, Kasuga-koen 6-1, Kasuga, Fukuoka 816-8580, Japan; 2 Institute of Innovation for Future Society, Nagoya University, Furu-cho, Chikusa, Nagoya, Aichi 464-8603, Japan; 3 School of Mechanical Engineering, Pusan National University, Geumjeong-gu, Busan 46241, South Korea; 4 Research Center for Next Generation Refrigerant Properties (NEXT-RP), International Institute of Carbon-Neutral Energy Research (WPI-I2CNER), Kyushu University, 744 Motooka, Nishi-ku, Fukuoka, Fukuoka 819-0395, Japan; 5 BK21 FOUR ERICA-ACE Center, Hanyang University, 55 Hanyangdaehak-ro, Sangnok-gu, Ansan, Gyeonggi-do 15588, Republic of Korea; 6 Department of Mechanical Engineering, Hanyang University, 55 Hanyangdaehak-ro, Sangnok-gu, Ansan, Gyeonggi-do 15588, Republic of Korea

**Keywords:** Computational chemistry, Materials science, Computational materials science

## Abstract

Physisorption of gases has been widely applied in thermal energy utilization and purification processes. Diffusion in porous media has been well studied. However, molecular-scale adsorbate diffusion mechanism remains unexplored. In this study, molecular dynamics have been employed to elucidate the diffusion behaviors of liquid and gaseous methane adsorbed in Cu-BTC (Copper(2+) 1,3,5-benzenetricarboxylate). Based on the energy distribution and trajectories of adsorbed molecules, a hypothesis is proposed that the adsorbed phase can be classified into four types: bound molecules (oscillate around a specific region of the adsorbent), generally adsorbed molecules (within the range of surface interaction and possess negative total energy), non-adsorbed molecules (within the range of surface interaction, but having positive total energy), and free molecules (beyond the range of surface interaction). To support this hypothesis, further simulation of methane adsorption in MOF-5 (Zn_4_O(BDC)_3_) has been conducted and compared with existing experimental data, indicating the hypothesis has broader applicability.

## Highlights

• Adsorbed-phase transition can be indicated by specific heat capacity change
• The trajectory data revealed that some molecules are trapped in specific regions
• A hypothesis classifies adsorbed molecules by trajectory and energy distribution
• Energy distribution is used to distinguish between absolute and excess adsorption

## Graphical abstract

**Figure:**
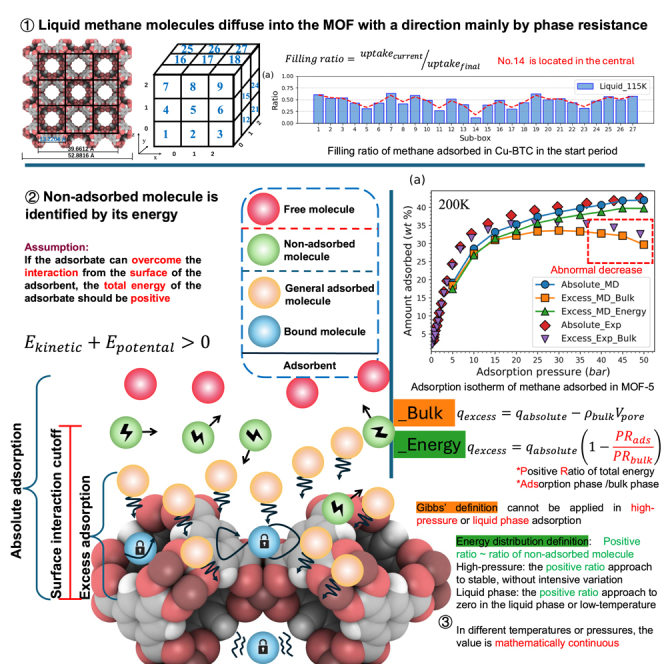

## Introduction

Physical adsorption technology, due to its simple principles and regeneration ability, has a wide range of applications in industry and environmental engineering field.^1^^,^^2^^,^^3^ For example, in the hydrogen fuel cell vehicle industry, hydrogen storage through adsorption not only provides high hydrogen density but also requires lower pressures compared to traditional compressed hydrogen storage, thus offering enhanced safety and has attracted considerable attention.^4^^,^^5^^,^^6^ In thermal energy utilization, adsorption heat pumps can utilize low-grade heat sources to drive cooling systems in buildings or raise the temperature of low-temperature heat sources to meet broader industrial needs.^7^^,^^8^^,^^9^ In environmental purification, adsorption technology holds promises for removing carbon dioxide from air or flue gas, making it a crucial component of carbon capture, utilization, and storage (CCUS).^10^^,^^11^^,^^12^ The development of physical adsorption technology is inseparable from advanced adsorbent synthesis technologies in recent years.^13^^,^^14^ Metal-organic frameworks (MOFs)^15^^,^^16^, zeolites,^17^^,^^18^ activated carbon,^19^^,^^20^^,^^21^ and other porous materials, with their high porosity, large specific surface area, and good thermal stability, have become ideal adsorbents.^22^^,^^23^

Current research on adsorption process characterization mainly focuses on adsorption capacity and isosteric heat.^24^^,^^25^ Four models primarily describe adsorption capacity: the Langmuir model for monolayer adsorption,^26^ the Brunauer-Emmett-Teller (BET) model for multilayer adsorption,^27^ the Dubinin-Radushkevich (DR) model for adsorption in porous materials,^28^ and Toth model for single component with ununiform phase adsorption.^29^ The isosteric heat model is based on the Clausius-Clapeyron relation.^30^ As an important parameter in the adsorption heat model, the density of the adsorbed phase currently lacks accurate measurement and calculation methods under experimental conditions.^31^^,^^32^ Yu et al. discussed the impact of methane adsorbed-phase density on isosteric heat in shale and proposed a constrained model based on the Langmuir-Freundlich adsorption model to limit the variation of adsorbed-phase density, making it more consistent with physical meaning and experimental results.^33^ In molecular simulations, the Clausius–Clapeyron relation can be calculated through energy variations to obtain isosteric adsorption heat.^34^ Saren et al. used Grand Canonical Monte Carlo (GCMC) simulations to study the adsorption of carbon dioxide on graphite surfaces with different pore sizes and functional group modifications.^35^ They found that reducing pore size and the presence of functional groups can significantly increase the isosteric heat.

However, except the synthesis field,^36^^,^^37^ researches on the diffusion mechanism of gases within adsorbents remains quite fundamental. Diffusion is mainly evaluated by obtaining the diffusion coefficient from adsorption kinetics curves and then calculating the activation energy using the linear relationship of temperature variation through the Arrhenius equation.^38^^,^^39^^,^^40^^,^^41^ There is a lack of detailed analysis on diffusion pathways or diffusion patterns within adsorbents.^42^ Both adsorption isotherm and kinetics measurements are indirect methods: either using manometric or gravimetric methods while detecting macroscopic changes in terms of pVT or mass. On the other hand, adsorption kinetics models invoke diffusion mechanisms inside the porous structures. These methods can assess the bulk or macroscopic behaviors rather accurately; however, they merely relate or cannot represent the physical phenomena at the molecular level. Collective behaviors of the dominating event might lead to the macroscopic properties. Nevertheless, it is important to examine the detailed mechanisms of the molecular clusters with respect to their energy levels. Such an approach can lead to a better understanding of the adsorption mechanism including the non-dominating phases or activities. Moreover, it is envisaged that adsorption as extreme conditions such as liquid phase for low critical temperature fluids such as methane might exhibit unconventional characteristics, which are not investigated in detail yet. In a broader sense, the concept of absolute and excess adsorption amount has mainly been explained from the perspectives of pore volume.^43^ However, the energy distributions of the molecules involved in the adsorption process have not been attempted to explain this concept.

To fill this important scientific gap, we aim to investigate the diffusion mechanism of various phase states of Lennard-Jones (LJ) fluid in MOFs through molecular dynamics (MD) simulations and identify diffuse mechanism. MD can record the atomic trajectories at each moment, making it easier to observe the detailed diffusion behavior inside the pores. The properties of LJ fluid are fundamental and intuitive, best reflecting the common characteristics of fluids. We chose MOFs because of their regular structure, compactness (pore sizes smaller than the cutoff), and their relative complexity compared to the activated carbon model of double-layer graphite,^44^^,^^45^ which allows deeper investigation.

In this paper, we first use MD to simulate the adsorption of LJ fluid methane in Cu-BTC under low-temperature liquid, low-temperature gas, and room temperature gas conditions. We compare the adsorption kinetic curves, kinetic energy of adsorbed molecules, and heat capacity. Based on the trajectory data, we perform statistical analysis of the diffusion paths of methane molecules in the MOF under these three conditions. Furthermore, using the radial distribution function (RDF), we infer the adsorbed phase’s state.

We propose a hypothesis that the adsorbed molecules can be classified into four types according to MD: bound molecules, generally adsorbed molecules, non-adsorbed molecules, and free molecules. To further explain and support this hypothesis, we discuss the relationship between the absolute adsorption and excess adsorption. We simulate the adsorption process of methane in MOF-5 and compare with existing experimental results. We find that evaluating absolute and excess adsorption using the concept of energy distribution can explain the phenomenon of the absence of free molecules in low-temperature and liquid-phase adsorption, whereas the traditional Gibbs excess adsorption formula does not apply. This paper offers several new perspectives that will contribute to the understanding of physical adsorption phenomena at the molecular level.

## Results

### Overall adsorption behaviors of methane within Cu-BTC

This section explores the overall adsorption process, revealing the variations in adsorption capacity, heat capacity, total kinetic energy, and the average kinetic energy of individual molecules over time.

**Figure 1:**
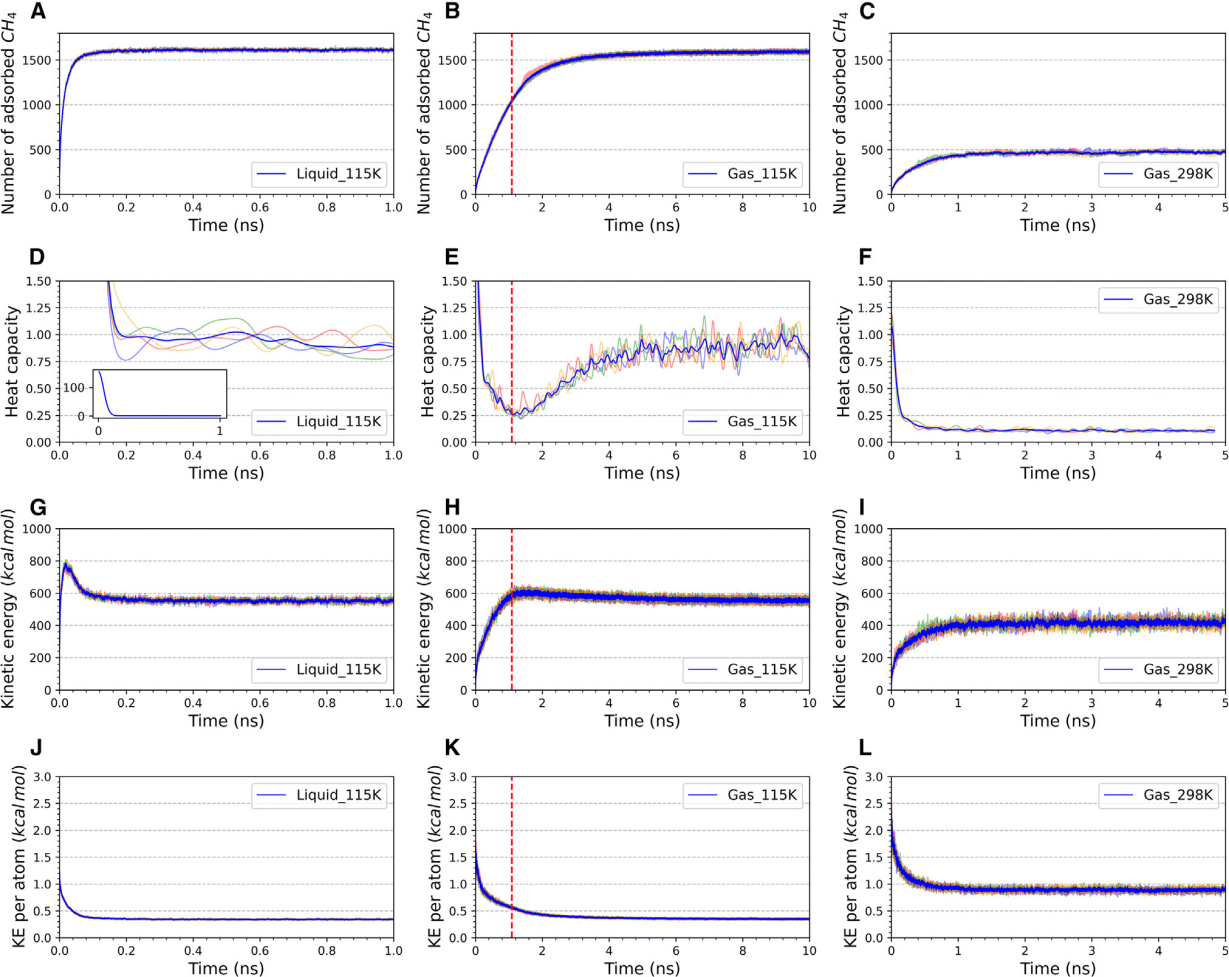
Physical properties of adsorbed methane within Cu-BTC under the bulk phase of low-temperature liquid, low-temperature gas, and room temperature gas (A–C) Number of adsorbed molecules, (D–F) heat capacity, (G–I) kinetic energy, (J–L) kinetic energy per atom.

Figure 1 shows the variations of different physical properties of adsorbed methane molecules within the Cu-BTC framework under different bulk phases during adsorption process simulations. From left to right, these represent low-temperature liquid adsorption, low-temperature gas adsorption, and room temperature gas adsorption. The results of four independent simulations are shown in light colors, with the average values represented in dark blue. First is the adsorption kinetic curve of methane. From Figures 1A and 1B, we can see that at the same temperature, the uptake of liquid-phase adsorption is only 0.5% higher than that of the gas phase, suggesting that Cu-BTC has reached its maximum adsorption state at this temperature and does not vary with pressure. They are at the same temperature, but the liquid environment is at a higher pressure, so the adsorption rate of the liquid phase is much higher than that of the gas phase. Figure 1C shows the room temperature gas adsorption, where the uptake is much lower than in low-temperature adsorption states. To observe the change in adsorbed-phase behavior in these cases, we calculated the heat capacity during the adsorption process in the energy fluctuation formula (Equation 1 for Figures 1D–1F)^46^:

(Equation 1) $$C=\frac{\left\langle E^{2}\right\rangle -{\left\langle E\right\rangle }^{2}}{{mk}_{B}T^{2}}$$

where *C* is the heat capacity, *E* is the total energy, m is the molecule mass, *k*_*B*_ is the Boltzmann constant, and *T* is the simulation temperature. In molecular simulations, the calculation of heat capacity using energy fluctuations can be affected by sampling intervals and size effect,^47^^,^^48^ which introduces numerical errors. The purpose of the heat capacity calculation here is to support the assessment of changes in the adsorbed phase, so we normalized and Gaussian-smoothed the data using the heat capacity of liquid-phase adsorption as a reference line. In the low-temperature liquid and room temperature gas adsorption cases (Figures 1D and 1F), the heat capacity initially decreases significantly and then stabilizes as adsorption approaches equilibrium. However, in low-temperature gas adsorption (Figure 1E), the heat capacity decreases first, then increases, and finally stabilizes. We hypothesize that this turning point marks the occurrence of a phase change. A vertical red auxiliary line is drawn at the turning point, which corresponds to the point where the adsorption rate decreases in Figure 1B, indicating a shift in the adsorption stage.

Next, the overall kinetic energy changes were examined (Figures 1G–1I). The overall kinetic energy during the low-temperature liquid and gas adsorption stages first increases and then decreases, showing a trend similar to “car braking.” In contrast, the overall kinetic energy of room temperature gas adsorption increases continuously until saturation. It is related to the adsorption temperature. After comparing the energy changes under NVE conditions, the main reason for “braking” is the temperature control. At the beginning of adsorption, the Cu-BTC framework is empty, and the potential energy difference causes methane molecules to accelerate into the framework, increasing their velocity compared to the bulk phase. After the methane molecules are adsorbed, their potential energy decreases, and part of the energy acts as kinetic energy, transferred to other methane molecules. Without temperature control, the input of energy transfer would increase the overall temperature of the adsorbed methane, known as adsorption heat. In NVE simulations, the temperature of adsorbed methane can reach 142 K in a 115 K environment, and 307 K in a 298 K environment. The thermostat in NVT forces some kinetic energy to be transferred away of the simulation box. The proportion of energy transferred is larger in low-temperature simulations, which leads to a decrease in overall kinetic energy—this is the result of forced velocity correction. In 298 K, the energy transfer is relatively mild, so there is no downward trend. Despite the influence of temperature control, in Figure 1H, we can see that the auxiliary line also falls precisely at the turning point of kinetic energy change, indicating that a phase transition in adsorption stages indeed occurred. Combined with the changes in heat capacity, a phase change is a reasonable explanation.

Figures 1J and 1L show the average kinetic energy changes of each adsorbed methane molecule. In the beginning, the average kinetic energy differs due to the differences in the bulk phase. Under the same temperature, the liquid phase is more stable than the gas phase. Under different temperatures, a higher temperature has a higher average kinetic energy. After adsorption, the average kinetic energy of the molecules decreases, consistent with physical sense. Additionally, the fluctuation of the kinetic energy curve varies under the three bulk conditions, with the lowest fluctuation in low-temperature liquid adsorption and the most intense in room temperature gas adsorption. To further analyze the differences in adsorption mechanisms, we conducted local statistical analyses of Cu-BTC.

### Local adsorption behaviors of methane with Cu-BTC

This section focuses on the diffusion behavior of methane molecules within each cage of the Cu-BTC framework, providing a detailed analysis of localized adsorption dynamics.

**Figure 2:**
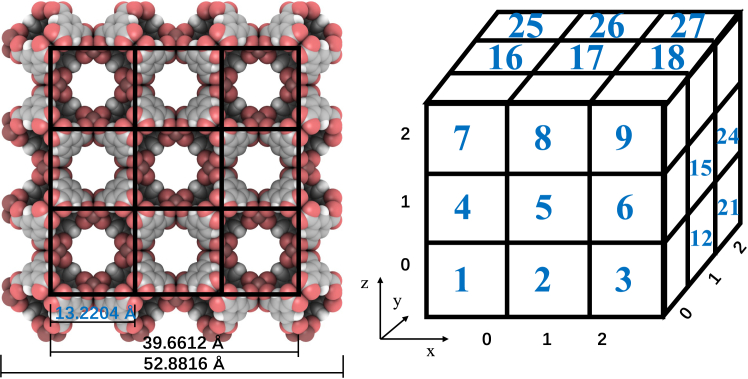
The schematic of sub-boxes separation

**Figure 3:**
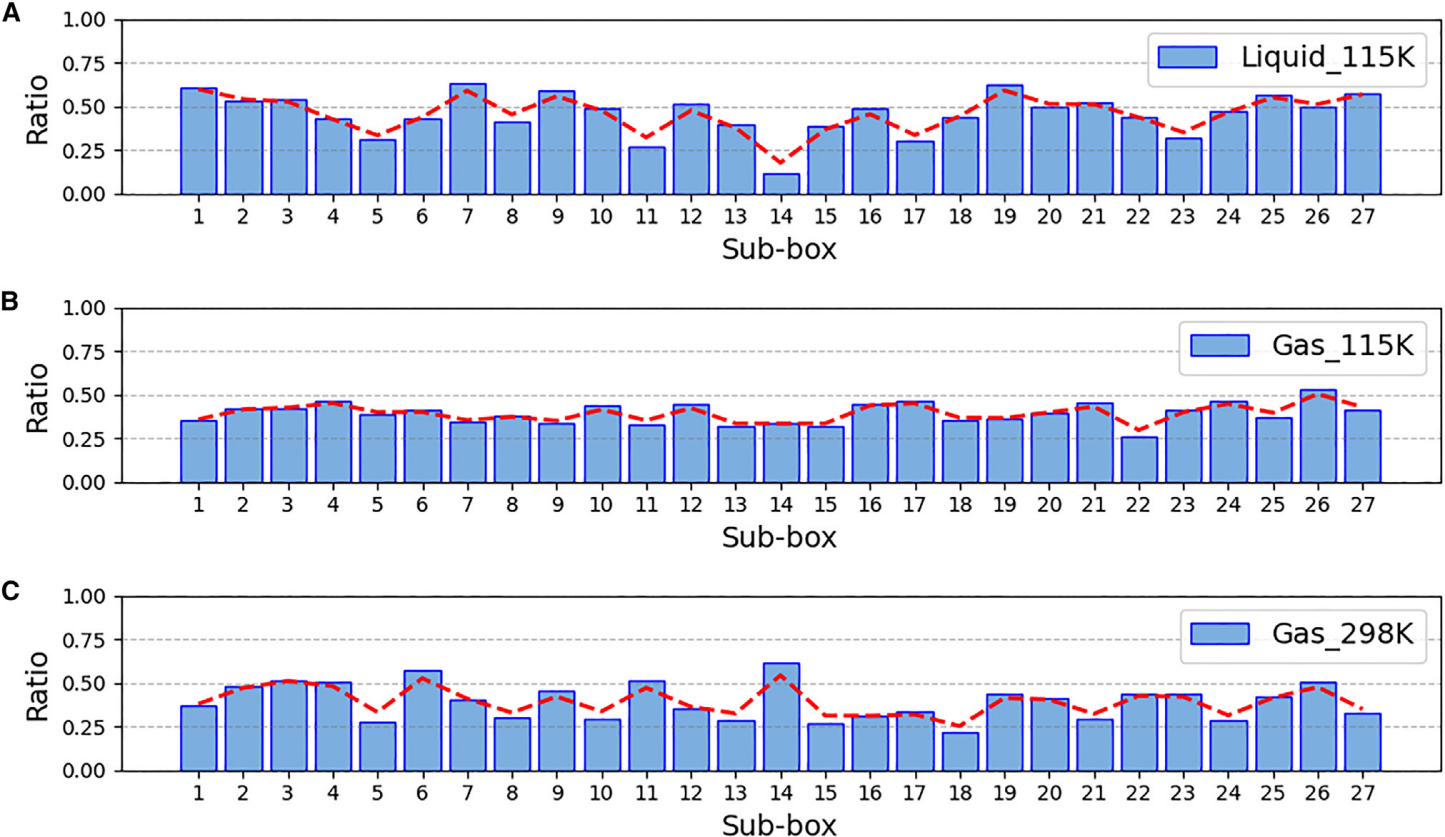
Filling ratio of methane adsorbed in Cu-BTC in the start period (A) Filling ratio under low-temperature liquid, (B) filling ratio under low-temperature gas, and (C) filling ratio under room temperature gas.

As shown in Figure 2, the 2-2-2 Cu-BTC unit cell, excluding the surface structure, the part outside black line, contains 3 × 3 × 3, 27 cubic cages in total. Each cage is in the length of 13.2204 Å, one-fourth of the Cu-BTC, duo to its periodic structure. By using cubes to encapsulate each cage, we obtained 27 sub-boxes. Based on the trajectory data obtained from the adsorption process simulation, we recorded the changes in adsorbed molecule number, kinetic energy, and potential energy in each sub-box over time. The variation trends of physical properties in each sub-box were similar to the overall trends, but the fluctuations in the curves reflect molecular exchanges between the sub-boxes (see Figures S1–S9). Methane molecules in low-temperature liquid adsorption were the most stable, whereas those in room temperature gas adsorption were the most active. Additionally, in the initial stage of adsorption, the distribution of uptake differed across the three bulk phases, as shown in Figure 3.

Figure 3 shows the filling ratio of methane uptake at the start period in each sub-box compared with the final saturated condition under the three bulk phases, where the start period is defined as the time corresponding to 10% of the total saturation adsorption time. The saturation uptake here is the average uptake over the last 5% of the simulation time. Please note that sub-box 14 is located at the very center. From Figure 3A, we can see that in the low-temperature liquid adsorption, the adsorption is gradually filling from the outside into the inside, with a clear direction in diffusion. In the low-temperature gas adsorption (Figure 3B), molecules tend to diffuse into every corner of the cavity, and the uptake in each sub-box is relatively uniform. Finally, in the room temperature gas adsorption (Figure 3C), the molecule diffusion is very active, leading to a more random transient distribution.

To summarize the adsorption behavior under the three bulk phases: in the low-temperature liquid adsorption, although the kinetic energy of the molecule is low, the strong potential energy difference causes methane to quickly flow into Cu-BTC. After filling the surrounding cages, the center is filled at last. Due to the liquid adsorption, methane molecules remain tightly combined during the adsorption process, and molecular exchange between cages is very small. In low-temperature gas adsorption, the molecular kinetic energy is low, and the potential energy difference is much lower than in the liquid phase, causing methane molecules to enter Cu-BTC slowly. The diffusion within Cu-BTC lacks direction, but is gentler, filling each cage relatively uniformly. Once the uptake reaches a certain level, both the adsorption rate and molecular movement slow down, and a phase change begins, with density continuing to increase. Due to the gaseous environment, there is a moderate degree of molecular exchange between cages. In room temperature gas adsorption, molecular kinetic energy is higher, but the potential energy difference is lower than in the liquid phase, resulting in a faster adsorption rate compared to low-temperature gas. Diffusion within Cu-BTC is also non-directional, randomly filling each cage. Due to the high kinetic energy, molecular exchange between cages is very active after saturation is reached.

### Adsorbed-phase analysis

This section utilizes radial distribution function (RDF) analysis to characterize the state of the adsorbed phase. The RDF represents the ratio of the local density of particle *j* at different distances from particle *i* to the average density distribution. When *i* and *j* are the same type of particle, the RDF can be used to determine the phase state. For example, in a single-component simulation, if the CH_4_-CH_4_ RDF exhibits only one peak, the system is in a gaseous state. If multiple peaks are present without reaching zero, the molecular arrangement is more compact, indicating a liquid state. If multiple peaks are observed with valleys reaching zero, the molecular arrangement exhibits long-range order, characteristic of a solid state.

When *i* and *j* are different types of particles, the RDF can be used to analyze distribution arrangement. In this study, for instance, the Cu-CH_4_ RDF helps assess the adsorption layer formation of methane within Cu-BTC pores. Combined with the CH_4_-CH_4_ RDF, it further helps in analyzing the phase state of the adsorbed phase.

**Figure 4:**
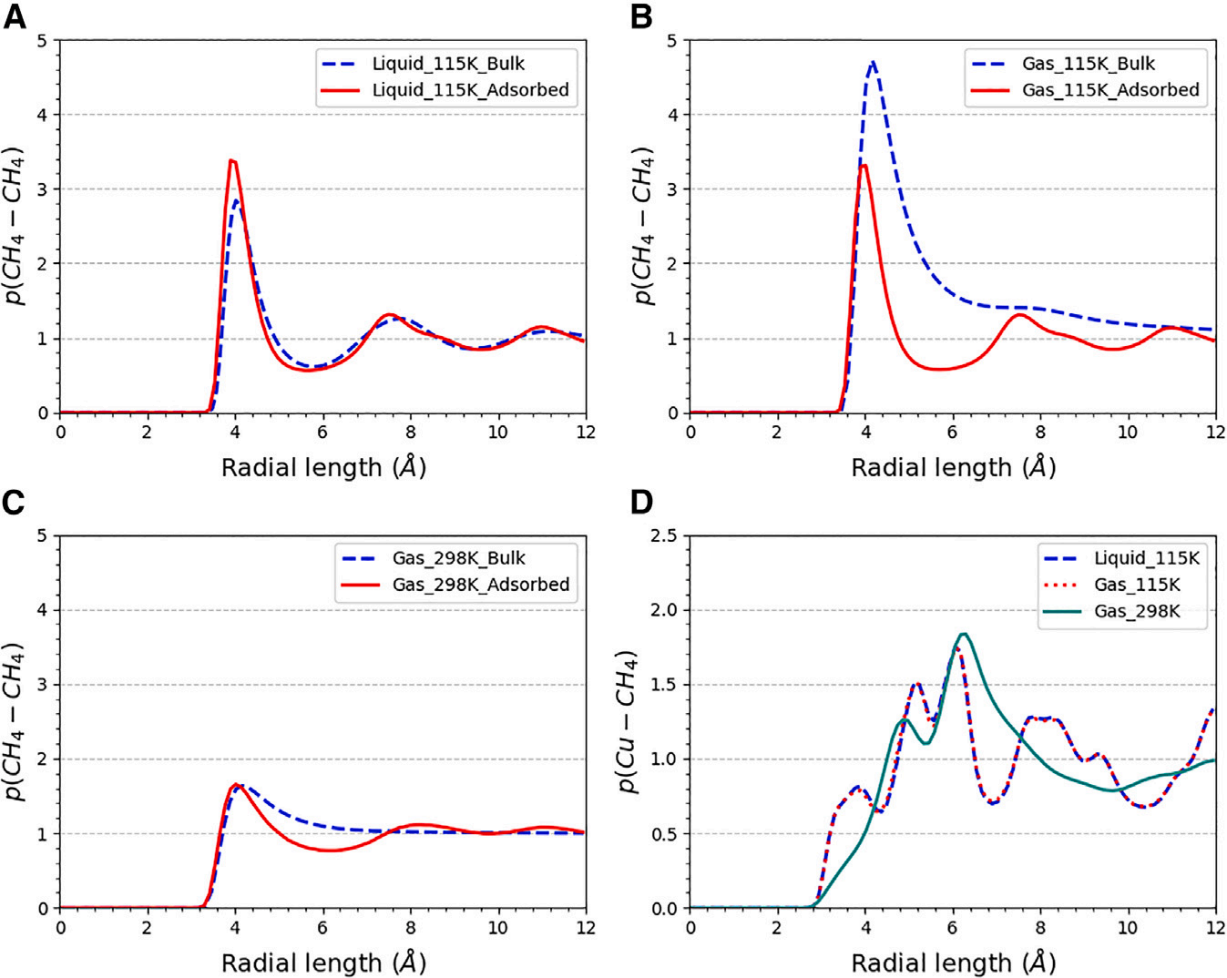
Radial distribution function of CH_4_-CH_4_ and Cu-CH_4_ (A) CH_4_-CH_4_ under low-temperature liquid, (B) CH_4_-CH_4_ under low-temperature gas, (C) CH_4_-CH_4_ under room temperature gas, and (D) Cu-CH_4_ under the three bulk phases.

The adsorbed phase at the saturation stage can be analyzed using the radial distribution function. Figure 4 shows the RDFs of CH_4_-CH_4_ and Cu-CH_4_ under different bulk phases. In Figures 4A–4C, it can be observed that for the bulk phase, the gas-phase RDF has only one peak, whereas the liquid-phase RDF has one main peak and two secondary peaks. In the simulation, the low-temperature and room temperature gas bulk phases were designed to have the same density, which means the same molecule number in the simulation box, so their peak values can be compared to some degree. The higher the peak value indicates the more densely methane molecules arrangement in a small range. Additionally, the low-temperature gas bulk-phase RDF shows a trend of forming a secondary peak, indicating a characteristic close to the liquid phase, as it is close to the triple point.

For the adsorbed phase, the uptake of low-temperature liquid and low-temperature gas adsorption is very close, reflected in nearly identical RDFs during the equilibrium adsorption simulation, with the third peak in the tail quickly converging to 1, displaying a standard liquid-phase characteristic. The adsorbed phase of room temperature gas adsorption also shows liquid-phase characteristics, but the main peak is not sharp. The structure of the MOF may affect the RDF distribution, leading to an uneven pattern, which could be mistakenly interpreted as a liquid phase.^49^

To further confirm the state of the adsorbed phase, the ratio of the adsorption mass to the pore volume (porosity = 0.7486) was used as the density, and together with the temperature, was input into the NIST database. The result indicated that the low-temperature adsorbed phase is in a two-phase state, whereas the room temperature adsorption corresponds to a supercritical state. The NIST data refer to pure substances and are only provided as a reference.

Figure 4D shows the RDF of Cu-CH_4_. At room temperature, there are only two relatively broad peaks, and the tail converges to 1, indicating that the distribution of methane molecules around copper atoms is relatively disordered, exhibiting gas-phase characteristics. At low temperatures, due to the higher uptake, methane molecules fill various regions. The RDF does not converge to 1 at the cutoff, indicating the formation of a complex and relatively regular distribution pattern. Additionally, there are no particularly broad peaks, suggesting that the adsorbed phase tends toward a liquid state. Based on this, we conclude that in the adsorption simulation, the adsorbed phase at low temperatures is in a liquid state, whereas the adsorbed phase at room temperature tends toward a gas-like supercritical state.

### Hypothesis on adsorbed molecules classification

By introducing the concepts of absolute adsorption and excess adsorption, this section proposes a hypothesis to classify adsorbed-phase molecules based on energy distribution and molecular trajectories.

After analyzing the adsorption modes and states of methane molecules, we aim to distinguish between absolute adsorption and excess adsorption in Cu-BTC. First, in the structure of Cu-BTC, the pore diameter is smaller than the cutoff of the LJ potential, meaning all adsorbed methane molecules are influenced by interaction from the MOF surface. Therefore, there are no free methane molecules, unlike in gas or liquid phases. J. Willard Gibbs proposed Gibbs excess adsorption theory that in gas-phase adsorption,^50^ the excess adsorption uptake is the absolute adsorption uptake minus the mass of the gas phase, where the gas phase is defined macroscopically, as shown in Equation 2:

(Equation 2) $$q_{excess}=q_{absolute}-\rho_{bulk}V_{pore}$$

where *q* is the uptake, ρ is the density, and *V* is the volume. In Equation 2, the free gas is considered as the product of its density and the pore volume, where the pore volume is typically determined using a helium probe molecule.^51^ The choice of probe molecule also affects the measured pore volume.^52^^,^^53^ However, previous molecular simulation studies have suggested that adsorbates are not uniformly distributed within the pores due to the presence of adsorption layers, meaning that the pore volume alone may not accurately represent the volume occupied by the free gas.^54^ From the analysis of the adsorbed phase above, it can be seen that room temperature gaseous methane exhibits a supercritical state after being adsorbed. Separating the gaseous phase from the supercritical state by the bulk-phase density is not rigorous.

Many studies have attempted to estimate the absolute adsorption amount or further calculate the density of the adsorbed phase based on the geometric distribution of the adsorbate.^54^^,^^55^ Here, we propose an alternative approach that utilizes the energy distribution of the adsorbate as a potential method for such estimations. We wonder that it can be distinguished via molecular motion. Conceptually, the gas-phase molecules in absolute adsorption should have enough energy to overcome the forces from the MOF surface and be able to move freely. In molecular simulations, this is represented by kinetic energy being greater than potential energy, meaning the total energy is positive.

**Figure 5:**
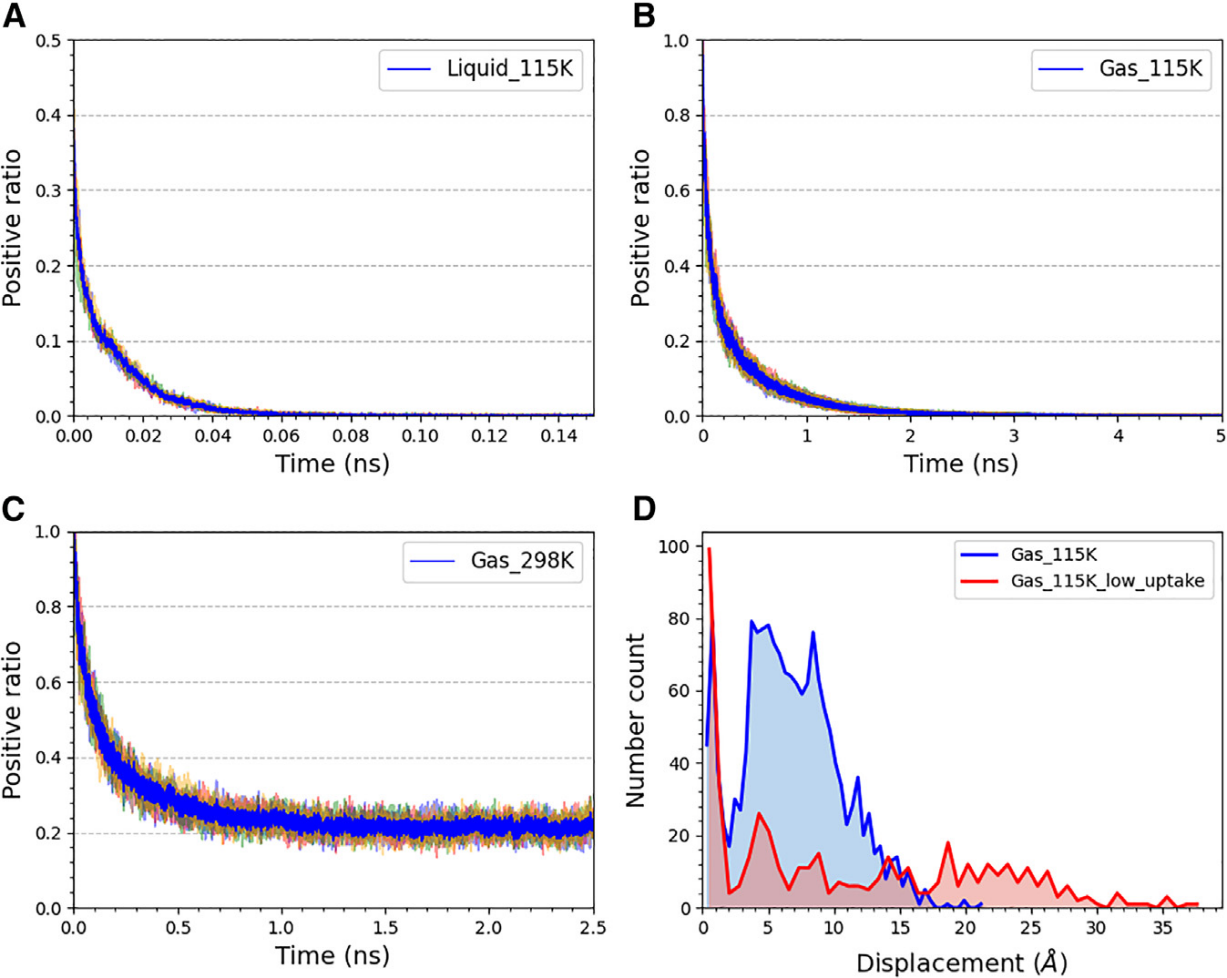
Positive ratio of total energy among adsorbed methane and displacement distribution (A) Positive ratio under low-temperature liquid, (B) positive ratio under low-temperature gas, (C) positive ratio under room temperature gas, and (D) the displacement distribution of adsorbed methane with 200 ps intervals under low-temperature gas-phase adsorption.

Based on this, we propose a hypothesis: the excess adsorption uptake is equal to the absolute adsorption uptake minus the number of free molecules with positive total energy. Following this idea, we calculated the proportion of methane molecules with positive total energy during adsorption, as shown in Figure 5. Figures 5A and 5B show the proportion of free molecules during low-temperature adsorption. It can be seen that after adsorption, the proportion of free molecules drops to 0%–0.1%. In other words, in the low-temperature liquid adsorbed phase, almost no free molecules exist, and the excess adsorption uptake is nearly equal to the absolute. This indirectly confirms that Gibbs excess adsorption is unsuitable under low-temperature conditions. Additionally, the proportion of free molecules in the liquid and gas phases differs at the start of adsorption. In liquid-phase adsorption, only a portion of molecules have positive total energy due to their close interactions. In gas-phase adsorption, the initial proportion is 100%, also shown in Figure 5C. In room temperature gas adsorption, when adsorption reaches saturation, 23% of the adsorbed molecules possess positive total energy. We believe this proportion represents the difference between absolute adsorption and excess adsorption under these conditions.

Likewise, we assume that bound molecules are those that cannot escape the interaction forces of the MOF surface and wander in place for a certain period. Following this idea, we used OVITO to calculate the displacement of molecules. The displacement of a molecule is the absolute value of the distance between its position at a given time and its position at the previous time. The formula is shown below:

(Equation 3) $$D=\left|r_{t}-r_{0}\right|$$

where *D* is the displacement, and *r* is the coordinate of a molecule. Here, we cannot use the trajectory data from the adsorption process simulation, as the number of molecules changes over time. Therefore, we used the trajectory data from the equilibrium adsorption simulation for this analysis.

Since molecules are moving constantly, displacements over short time intervals do not reveal significant movement characteristics, but a specific long-time interval might offer new insights. The blue line in Figure 5D shows the displacement distribution of adsorbed methane with 200 ps intervals under low-temperature gas adsorption. A sharp peak can be seen in the region below 2 Å, whereas the peak on the right tends to follow a normal distribution. We speculate that this sharp peak corresponds to the number of trapped methane molecules. To further confirm this, we conducted another equilibrium adsorption simulation at 115 K with reduced uptake (equivalent to the uptake in high-temperature gas adsorption). The displacement at 200 ps intervals is represented by the red line. Similarly, a sharp peak appears around 2 Å, suggesting that this phenomenon is not accidental.

After confirming the visualized trajectory data (see Figures S10–S13), we found that these methane molecules are distributed near the copper atoms or within the joints connecting the cages, wandering around a certain point or within a specific region. Besides, the movement of methane molecules is more unrestricted in reduced uptake, so the range of displacements is broader than blue. This situation also exists in room temperature simulation, only around 9.2% of molecule has such behavior in the shorter interval of 10 ps, indicating that temperature is an important factor.

Based on this discovery, the previous hypothesis can be further refined. In physical adsorption, based on the energy distribution and the molecule trajectory, they can be divided into four categories, from strongest to weakest: The first category is **bound molecules**. Due to the structure of the adsorbent and the temperature, these molecules wander around a specific location, unable to move freely. The second category is **generally adsorbed molecules**. Although they cannot escape the influence of surface interaction, their movement is not restricted. The third category is **non-adsorbed molecules**. These molecules, although within the range of surface interaction (within the cutoff), have enough energy to escape and move relatively freely. The fourth category is **free molecules**, which are far from the adsorbent surface (beyond the cutoff), with completely unrestricted movement, exhibiting bulk-phase behavior.

### Validation through methane adsorbed in MOF-5

This section modifies and validates the proposed hypothesis through methane adsorption simulations in MOF-5, demonstrating its applicability and robustness in different adsorption systems.

The total energy distribution can be referenced from the simulation results of bulk-phase methane molecules. The total energy of the low-temperature liquid is mostly negative, meaning that only a very small number of molecules can escape from the surface. Physically, the phenomenon of molecules escaping from the liquid is evaporation, which happens slowly at low temperatures, consistent with real-world conditions. In the low-temperature gas phase, the ratio of positive to negative total energy is 7:3, meaning that a small portion of gas molecules undergo liquefaction at low temperatures, forming small clusters of molecules as droplets. At room temperature, molecules almost have positive total energy, showing gas-phase features.

Temperature has a significant effect on adsorption capacity. Higher temperatures mean higher kinetic energy of the adsorbate molecules, making it easier for them to overcome the potential energy barrier of the adsorbent surface, which corresponds to having a higher number of adsorbed molecules with positive total energy. Temperature also affects the adsorbed phase. Compared to the bulk phase, the internal space of the adsorbent acts as an ultra-high negative pressure region. Thus, near the phase change temperature, even gas molecules entering the adsorbent may undergo liquefaction. The total energy of liquid-phase molecules is mostly negative, further preventing them from escaping, thereby increasing the uptake.

**Figure 6:**
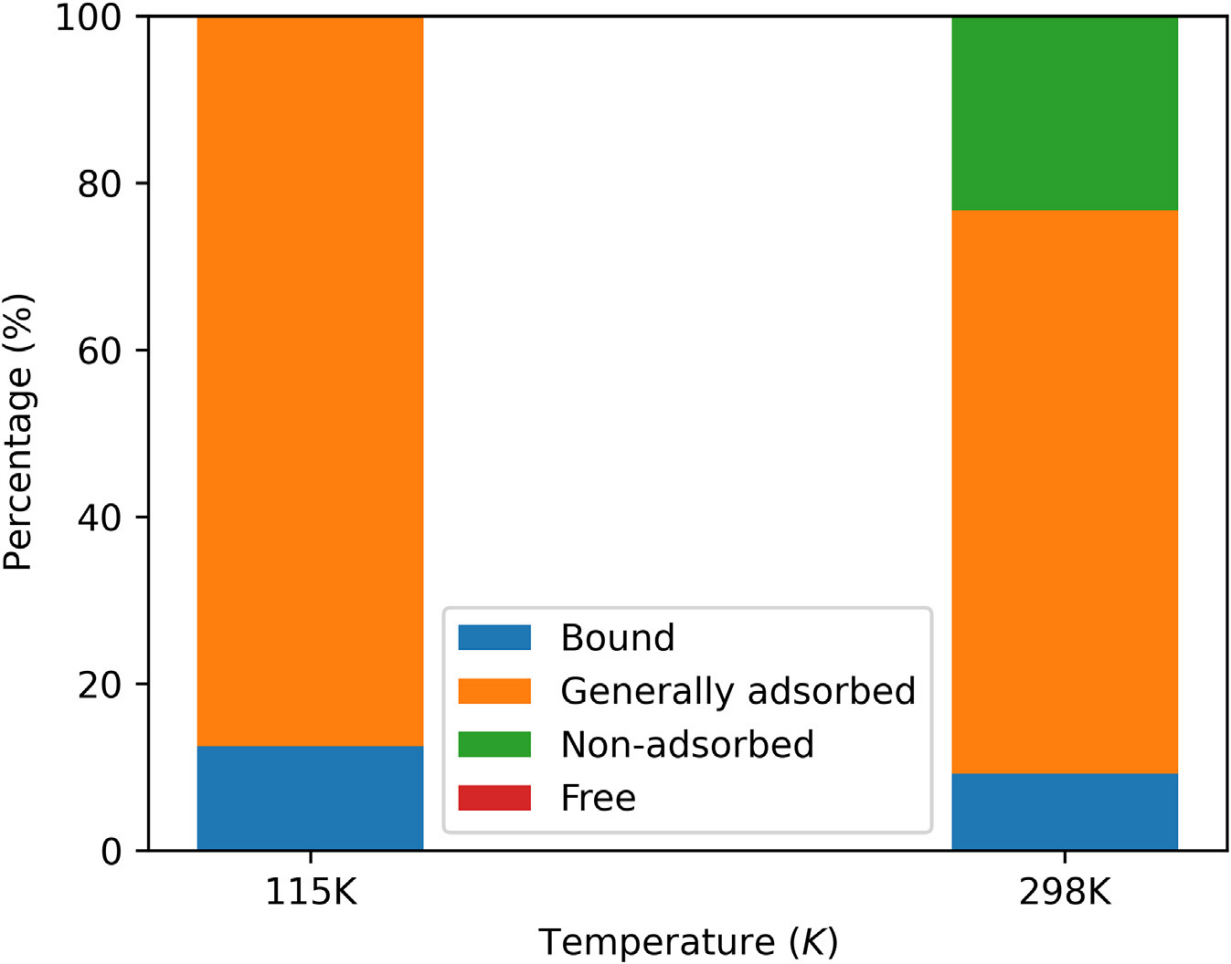
Adsorbed-phase classification of methane in Cu-BTC

The total energy distribution of the bulk phase could perhaps be used as a correction factor for adjusting excess adsorption, so that the amount of non-adsorbed can also be calculated, as in Equations 4 and 5:

(Equation 4) $$q_{excess}=q_{absolute}\left(1-\frac{R_{ads}}{R_{bulk}}\right)$$

(Equation 5) $$q_{non-adsorbed}=q_{absolute}\left(\frac{R_{ads}}{R_{bulk}}\right)$$

where *R* is the positive ratio of total energy for methane molecule, and *ads* denotes the adsorbed phase. After the modification, the classification of adsorbed phase is described in Figure 6.

The bound molecule is obtained from displacement distribution. As mentioned above, the interval in 115 K is 200 ps, whereas 10 ps in 298 K. The non-adsorbed molecule is mortified by Equation 5. It can hardly exist in low temperature, since in the liquid adsorbed phase, the total energy of molecules is almost negative. However, it is opposite in high temperature. In Cu-BTC, the pore diameter is 13.2204 Å in shown in Figure 2, where the radius is shorter than the cutoff, thus there is no free molecule. In that case, the rest are generally adsorbed molecules. Besides, not only the temperature but also the pressure is also an important factor influencing the distribution of particle energies.

**Figure 7:**
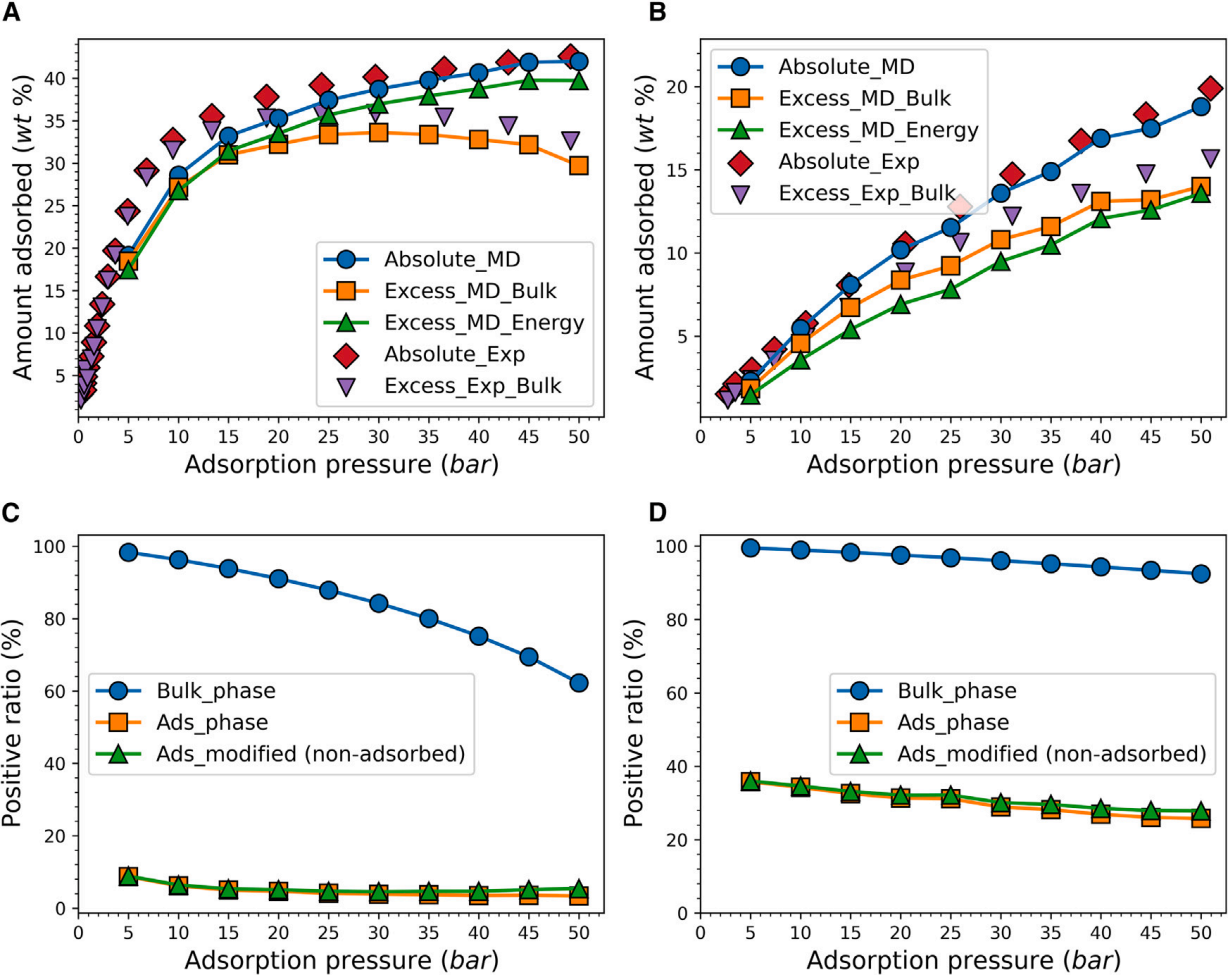
Adsorption result of methane adsorbed in MOF-5 Adsorption isotherm of methane adsorbed in MOF-5 at (A) 200 K and (B) 300 K. The positive ratio of the total energy of methane molecules at (C) 200 K and (D) 300 K.

To further clarify the effect of pressure, we additionally performed adsorption simulations of methane in MOF-5 at 200 K and 300 K with pressures ranging from 5 bar to 50 bar and compared them with the experimental results of Zhou et al.^56^ Figures 7A and 7B show the adsorption isotherms from both simulation and experiment, with the uptake converted to the mass ratio of adsorbate to adsorbent, consistent with the experimental results. For absolute adsorption uptake, the simulated results at 300 K match the experiment well, whereas at 200 K, they are a bit lower than the experimental values in the low-pressure range.

For excess adsorption, it was calculated by Gibbs excess adsorption as in Equation 2 in the experiment, where the amount of free gas is related to the bulk gas density. In that case, the simulation and experimental trends are consistent. Since the porosity in the simulation is 0.8037^57^ and in the experiment it is 0.66, the simulation results are generally lower than the experimental ones. At 200K, as the pressure gets higher, the absolute adsorption uptake shows logarithmic growth, weaker than the linear growth of bulk gas density. Thus, the excess adsorption amount tends to decrease at high pressure.^58^ At 300 K, the trend of increasing absolute adsorption remains significant, thus the excess adsorption also increases accordingly.

From the energy distribution perspective, non-adsorbed molecules are considered to be the set of molecules whose energy distribution is similar to that of the bulk phase. In the simulation, increasing pressure decreases the proportion of particles with positive total energy in both the adsorbed phase and the bulk phase, as shown in Figures 7C and 7D. However, after modification, the calculated proportion of non-adsorbed molecules decreases with increasing pressure and then tends to stabilize at a certain value. This proportion also decreases with decreasing temperature. Combined with the energy changes observed in the adsorption process for Cu-BTC, we find that determining non-adsorbed molecules based on energy distribution can well explain the absence of a distinction between absolute and excess adsorption in liquid adsorption. This is because, at low temperatures, the total energy of the adsorbed phase is almost below zero, indicating a liquid state with almost no free molecules, indicating that the Gibbs excess adsorption does not apply.

Currently, the energy distribution is mainly provided by MD, and direct observation in experiments remains challenging. If energy consumption during the desorption process could be measured precisely in the future, it might support this viewpoint of energy distribution.

### Classification of bound molecules

The final section addresses the issue of classifying bound molecules, providing a detailed discussion of the criteria and methodologies utilized.

The bound molecules in the adsorption classification can be compared to the boundary layer in fluid mechanics. A more accurate description would be a boundary region, and the determining factor for this boundary region is the adsorption site. Here, the determination of adsorption sites is not based on the number of adsorbates around a specific atom but on the movement of the adsorbates. In that case, the adsorption sites can be anywhere (Figure S14). From the simulation results, we can observe that molecules in the boundary region exhibit periodic motion, where they can circle around and return to their original position. Currently, we find that temperature has a significant effect on these phenomena, because the velocities of molecules are different based on temperature, thus the period of circling around is affected. Meanwhile, the temperature affects the adsorption phase inside the adsorbent. Different adsorption phases might cause different potential distributions to affect the adsorbate movement as well. Moreover, the distribution of bound molecules is likely to vary significantly across different materials. For example, it is quite possible to have no bound molecules in the graphite layer since the surface is uniform. This concept needs further investigation.

## Discussion

In this paper, we first used MD to simulate the adsorption of LJ fluid methane in Cu-BTC under low-temperature liquid, low-temperature gas, and room temperature gas conditions. From the analysis of the adsorbed phase, it was found that adsorption at low temperatures exhibited liquid-phase characteristics, and a phase change process occurred during low-temperature gas adsorption. In the analysis of adsorption path, it was observed that during gas-phase adsorption, the adsorbate molecules first diffuse into various cavities. At low temperatures, the diffusion is more uniform, whereas at room temperature, the diffusion is more random. In liquid-phase adsorption, molecules diffuse with direction, moving from the outer region to the inner region. Additionally, during diffusion, the molecules tend to exhibit liquid-like behavior, as reflected in the low proportion of molecules with positive total energy at the initial stage of adsorption and the smooth molecular exchange between sub-boxes.

Secondly, regarding absolute and excess adsorption, we proposed a molecular-dynamics-based hypothesis for physical adsorption: in physical adsorption, based on the energy distribution and molecule trajectories, they can be classified into four categories, from strongest to weakest. The first category is **bound molecules**. Due to the structure of the adsorbent and the adsorption temperature, these molecules are restricted in their movement and remain near the same position for an extended period. The second category is **generally adsorbed molecules**. Although they cannot escape the surface forces, their movement along the surface of the adsorbent is not restricted. The third category is **non-adsorbed molecules**. Although they are within the range of intermolecular forces, their energy is high enough to escape the surface forces of the adsorbent, allowing relatively free movement. The fourth category is **free molecules**. These molecules are far from the surface of the adsorbent and exhibit unrestricted molecular motion.

To further verify this hypothesis, we conducted adsorption simulations of methane in MOF-5 at 200 K and 300 K and compared the results with experimental data. We found that if excess adsorption is calculated based on Gibb’s relations, which considered the density of the bulk phase, the simulation results are similar to the experiment. However, when excess adsorption is calculated based on the energy distribution perspective, the ratio of non-adsorbed and free molecules tends to stabilize as pressure increases, and this ratio decreases with decreasing temperature. This further supports the phenomenon of no distinction between absolute and excess adsorption in low-temperature liquid adsorption. This paper offers new perspectives and more concrete explanations for the understanding of the physical adsorption process.

### Limitations of the study

(1) **Condensation of gas molecules in porous materials:** it is a widely accepted consensus in physical adsorption that gaseous molecules condense after being adsorbed into porous materials. Typically, gaseous-phase molecules transition into a liquid-like or near-liquid state upon adsorption. However, whether this phase transition occurs at the gas-solid interface or within the solid’s interior remains unclear. In this study, the initial diffusion behavior of methane molecules in various sub-boxes provided insights into the gas-solid interface. Additionally, simulations of methane adsorption at low temperatures revealed a phase transition occurring within the solid adsorbent, as indicated by changes in heat capacity. Experimentally characterizing these findings would require measuring the distribution of adsorbates within the adsorbent every few seconds during real adsorption processes, which remains highly challenging at present.
(2) **Classification hypotheses of adsorbed phases:** from a mathematical perspective, the hypothesis based on energy distribution offers numerically continuous results across temperature and pressure variations, avoiding the unrealistic decrease in excess adsorption observed in the Gibbs excess adsorption theory at high pressures. However, this hypothesis is still in its initial stages and is derived from idealized MD simulations. It requires further critique and refinement from the scientific community.

## Resource availability

### Lead contact

Further information and requests for resources and reagents should be directed to and will be fulfilled by the lead contact, Haonan Chen (chn970127@gmail.com).

### Materials availability

This study did not generate new unique reagents.

### Data and code availability

• Data reported in this paper will be shared by the lead contact upon request.
• This study does not report original code.
• Any additional information required to reanalyze the data reported in this paper is available from the lead contact upon request.

## Acknowledgments

This research was supported by the 10.13039/501100003662Korea Evaluation Institute of Industrial Technology (KEIT) funded by the 10.13039/501100003052Ministry of Trade, Industry and Energy (MOTIE) of the Republic of Korea (NO. 20018410) and the 10.13039/501100003725National Research Foundation of Korea (NRF) funded by the 10.13039/501100014188Ministry of Science and ICT (MSIT) of the Republic of Korea (No. RS-2023-00259994).

## Author contributions

H.C., writing—original draft, writing—review & editing, conceptualization, methodology, simulation, validation, and investigation. S.S., writing—review & editing, methodology, and investigation. X.L., methodology, investigation, and formal analysis. J.H.J., supervision and investigation. T.M., supervision and investigation. Y.D.K., supervision, funding acquisition, and investigation. K.T., writing—review & editing, supervision, methodology, investigation, and conceptualization.

## Declaration of interests

The authors declare that they have no known competing financial interests or personal relationships that could have appeared to influence the work reported in this paper.

## Declaration of generative AI and AI-assisted technologies in the writing process

During the preparation of this work, the authors used ChatGPT-4o in order to improve the English vocabulary and readability. After using this tool/service, the authors reviewed and edited the content as needed and take full responsibility for the content of the publication.

## STAR★Methods

### Key resources table

**Table:** 

REAGENT or RESOURCE	SOURCE	IDENTIFIER
**Software and algorithms**		

LAMMPS	Open source	www.lammps.org

### Experimental model and study participant details

No experiments were conducted in this study.

### Method details

#### Molecule configurations and force field

All molecular dynamics (MD) simulations were conducted using the Large-scale Atomic/Molecular Massively Parallel Simulator (LAMMPS).^59^ The LJ particle, a coarse-grained methane, was modeled using the TraPPE model,^60^ and the force field was modified.^49^ The adsorbent, a 2 2 2 all-atom Cu-BTC supercell, had dimensions of 52.8816 Å and a density of 869.38 g/cm^3^. The LJ parameters of atoms in Cu-BTC were referred from the UFF and DREIDING force fields,^61^^,^^62^ the interactions between methane and Cu-BTC (in Table S1) were derived from Lorentz-Berthelot mixing rules,^46^ with a cutoff radius of 12.0 Å, no tail correction, and periodic boundary conditions. Verification of the force field, including checks for density, thermal conductivity, and adsorption isotherms, was conducted in our previous work.^49^ The size of Cu-BTC supercell was selected based on both the convergence of adsorption uptake and computational efficiency.

#### Simulation details

The adsorption process was divided into three stages: the bulk phase creation, a adsorption process simulation, and an equilibrium adsorption simulation, as shown in Figures S15A–S15C, visualized by iRASPA 2.3.3.^63^ Three bulk phases were considered: a gaseous phase at 25 bar and 298 K to simulate room-temperature gas adsorption, where the methane is dense enough to have statistical significance; a liquid phase at 25 bar and 115 K for liquid-phase adsorption, where close to triple point of methane and 25 bar can guarantee liquid phase; and a gaseous phase at 7 bar and 115 K for low-temperature gas adsorption. The low-temperature gaseous adsorption simulation was designed to observe phase change phenomena during adsorption, where the temperature of 115 K is slightly higher than the triple point of methane. The pressure of 7 bar was calculated, where its simulation box size, molecule number, and other simulation parameters were the same as those used in the 25 bar, 298 K room-temperature gas simulation, with only the temperature altered.

The simulation box was a 400 Å cube for the room-temperature gaseous phase, with 1 ns allocated for relaxation and another 1 ns for production. The box size for the liquid phase was 250 Å, with 200 ps for relaxation and production. All bulk phase creation simulations were conducted using the NVT ensemble, with a timestep of 1 fs and periodic boundary conditions, while the temperature was controlled using the Nosé-Hoover thermostat.^64^ The number of methane molecules was determined by the density values from REFPROP/NIST.^65^

The adsorption process simulation was restarted from the final state of the previous simulation. The Cu-BTC framework was inserted and frozen at the center of the simulation box, so that the framework was rigid and immobile. To avoid periodic boundary image errors, bond and angle forcefield information of Cu-BTC was removed, and methane molecules within that space were also deleted to ensure an initial adsorption uptake of zero. The main adsorption process was performed using the NVT ensemble with a timestep of 1 fs. The simulation durations were set to 1 ns for the low-temperature liquid phase, 10 ns for the low-temperature gas phase, and 5 ns for the room-temperature gas phase. During these simulations, the kinetic energy, potential energy, and number of adsorbed methane molecules were recorded. In addition, short NVT and NVE simulations were conducted under three bulk conditions for 0.15 ns, 2 ns, and 5 ns, respectively, to analyze the behavior of methane in the adsorbed phase. Coordinates, kinetic energy, and potential energy per methane atom were recorded every 20 timesteps. The NVE ensemble was utilized to avoid velocity modifications by the thermostat.

The equilibrium adsorption simulation was carried out to examine the behavior of the stable adsorption phase. It was restarted from the final state of the NVT non-equilibrium simulation, with the simulation box adjusted to the size of the Cu-BTC framework. The bond and angle forcefield data for Cu-BTC were reintroduced to allow for a flexible framework. The NVT ensemble was applied again, with a timestep of 1 fs, followed by 2 ns for relaxation and 2 ns for production.

#### Auxiliary simulation for methane adsorption within MOF-5

The simulation flow for MOF-5 and methane is similar to Cu-BTC and methane. The bulk phase is at two temperatures, 200 K and 300 K, and the ten pressures range from 5 bar to 50 bar. The adsorbent, a 2 2 2 all-atom MOF-5 supercell, had dimensions of 51.6640 Å and a density of 593.40 g/cm^3^ (Figure S15D). The interaction is described in Table S2, where the force filed of methane is validated by RASPA2.^66^^,^^34^ The only difference in the adsorption process simulation is the run time of the NVT ensemble, where for 5 bar to 30 bar is 10 ns, while for 35 bar to 50 bar is 5 ns.

### Quantification and statistical analysis

Each simulation involving Cu-BTC was independently repeated four times, and the average of the results was taken to ensure reliability. For MOF-5 was only one time, which is involved to verify absolute and excess adsorption. Post-processing was performed using JavaScript, Python, and OVITO 3.10.8.^67^
