# Supplementary material for: Diffusion mechanism and adsorbed-phase classification—molecular simulation insights from Lennard-Jones fluid on MOFs
Source: iScience. 2025 Mar 8;28(4):112181. doi: 10.1016/j.isci.2025.112181 (PMC11978323; doi:10.1016/j.isci.2025.112181)
Supplement: Document S1. Figures S1–S15 and Tables S1 and S2 [file mmc1.pdf]

iScience, Volume *28*

Supplemental information

Diffusion mechanism and adsorbed-phase classification—molecular simulation insights from Lennard-Jones fluid on MOFs

Haonan Chen, Sagar Saren, Xuetao Liu, Ji Hwan Jeong, Takahiko Miyazaki, Young-Deuk Kim, and Kyaw Thu

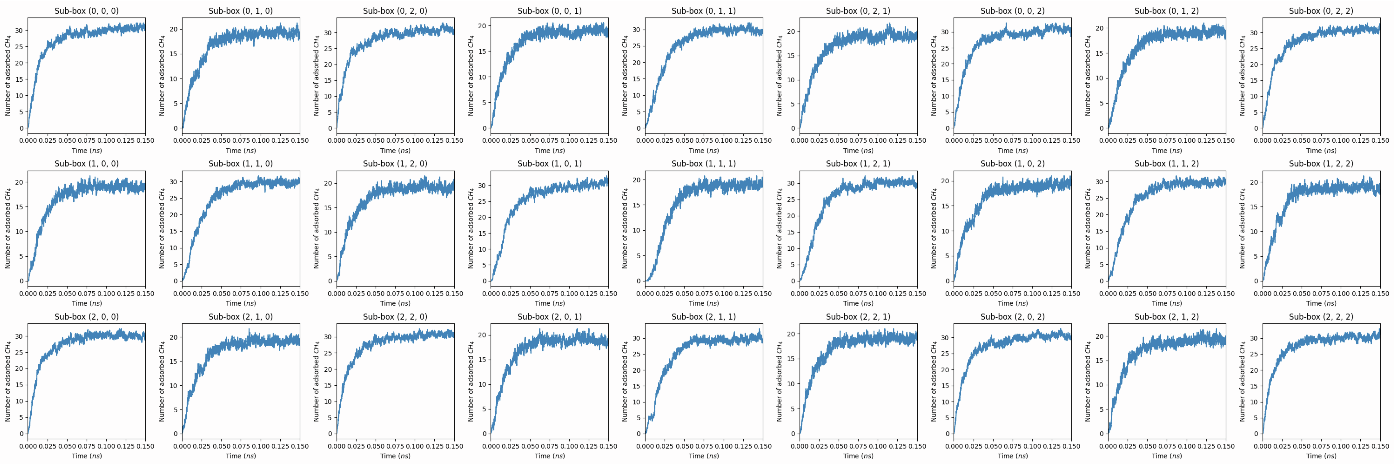

Figure S1. Number of adsorbed methane molecules within Cu-BTC under the bulk phase of low-temperature liquid, related to Figure 2. (Data are represented as the mean value)

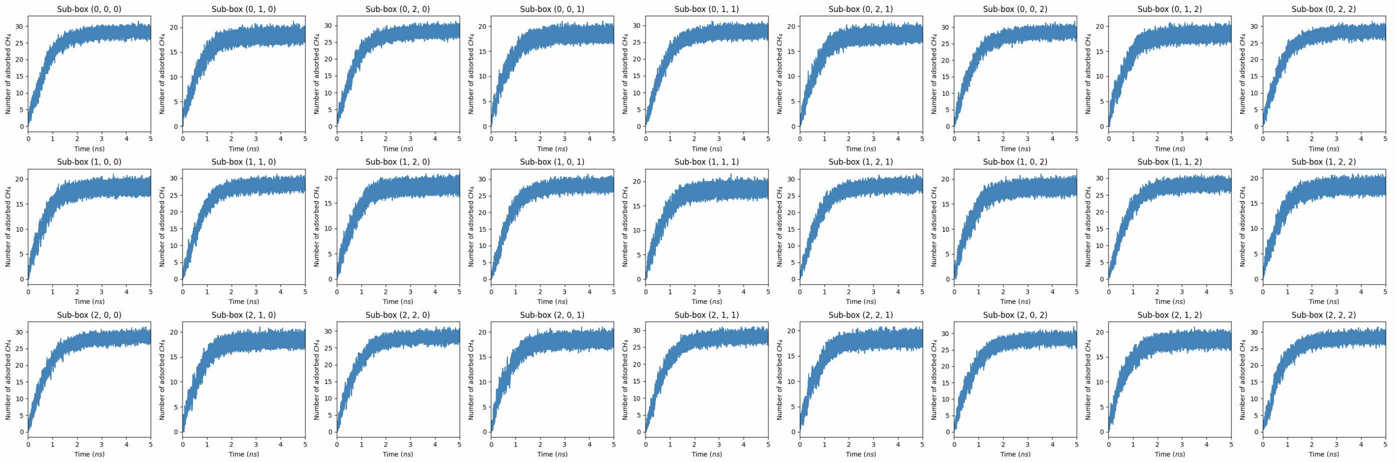

Figure S2. Number of adsorbed methane molecules within Cu-BTC under the bulk phase of low-temperature gas, related to Figure 2. (Data are represented as the mean value)

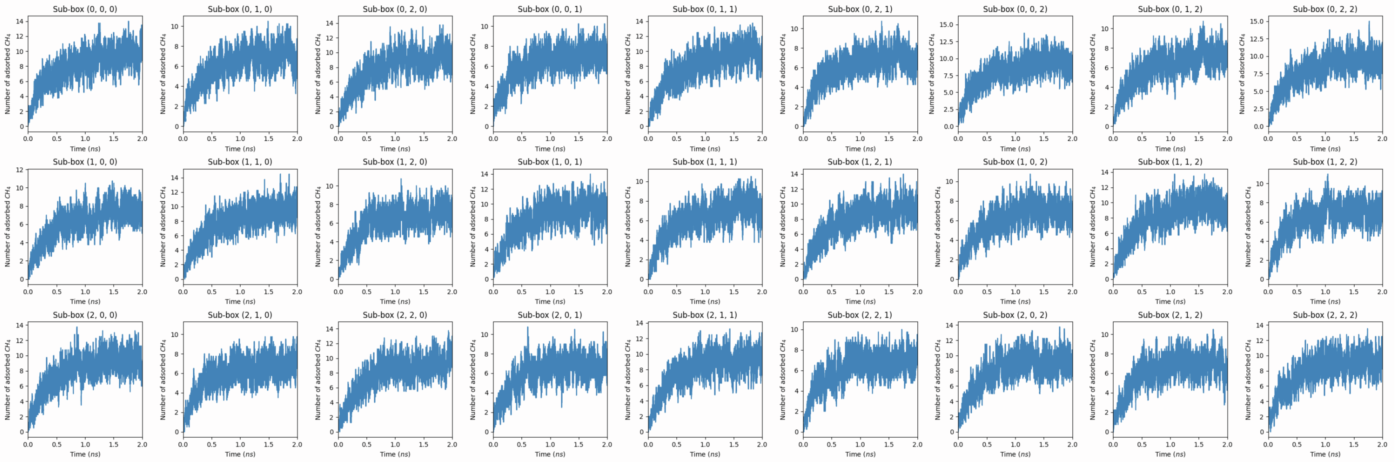

Figure S3. Number of adsorbed methane molecules within Cu-BTC under the bulk phase of room-temperature gas, related to Figure 2. (Data are represented as the mean value)

Figures S1-S2 describe the adsorption kinetics profiles in each sub-box according to three bulk phases. The trend of each profile is similar to the overall adsorption kinetics in Figures 1 a-c. However, the fluctuation of profiles differs from the bulk phase. The kinetics of low-temperature liquid adsorption is the most stable, while room-temperature gaseous is the most active.

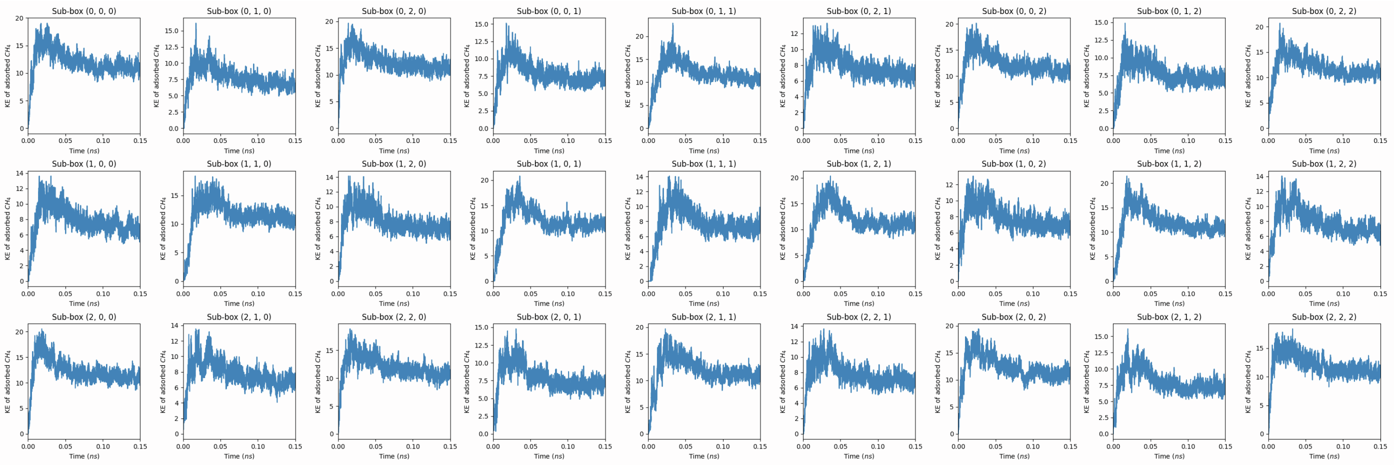

Figure S4. Kinetic energy of adsorbed methane molecules within Cu-BTC under the bulk phase of low-temperature liquid, related to Figure 2. (Data are represented as the mean value)

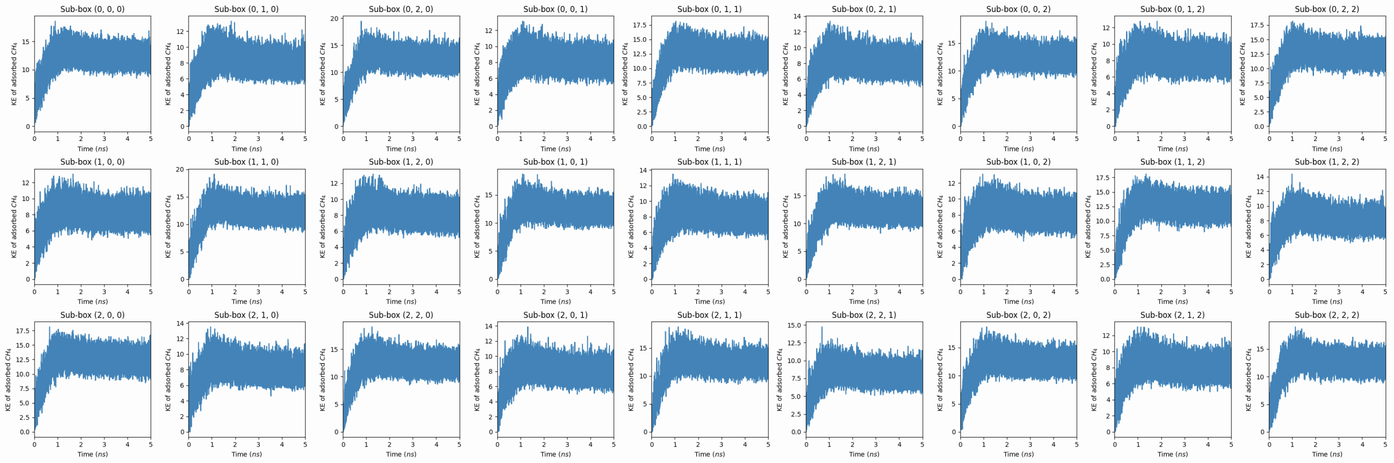

Figure S5. Kinetic energy of adsorbed methane molecules within Cu-BTC under the bulk phase of low-temperature gas, related to Figure 2. (Data are represented as the mean value)

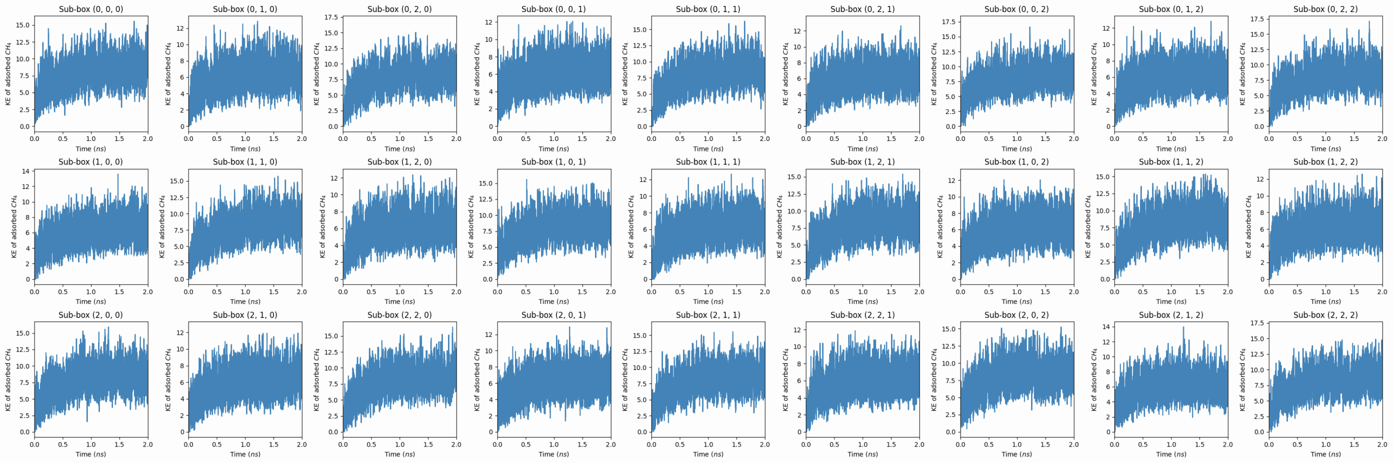

Figure S6. Kinetic energy of adsorbed methane molecules within Cu-BTC under the bulk phase of room-temperature gas, related to Figure 2. (Data are represented as the mean value)

Figures S4-S6 describe the sum of the kinetic energy of adsorbed methane molecules in each sub-box according to three bulk phases. The fluctuations of each profile indicate that the exchange of kinetic energy is more active in the gaseous phase adsorption than in the liquid phase.

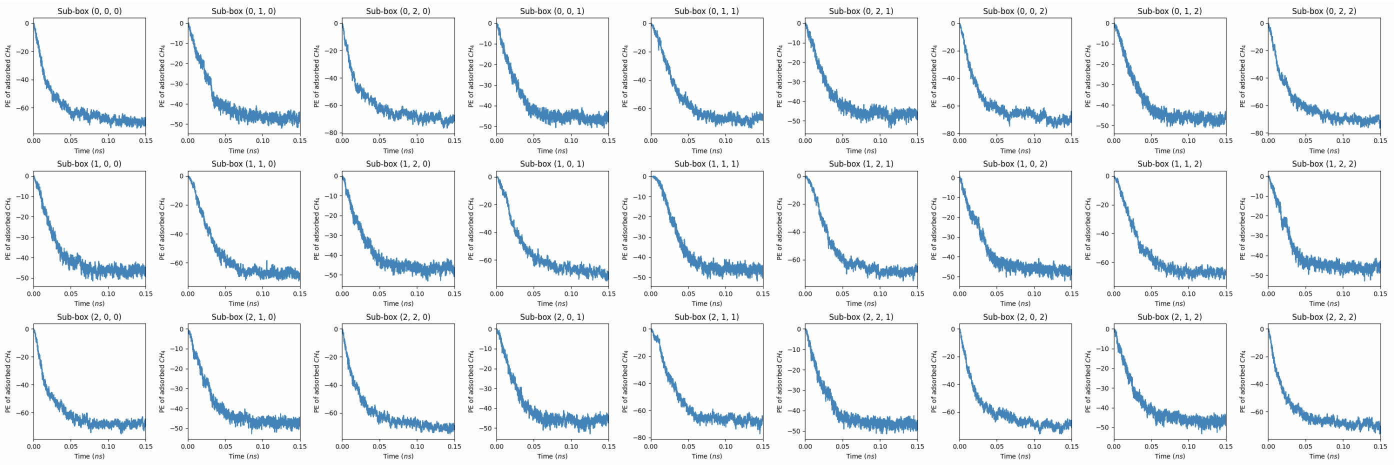

Figure S7. Potential energy of adsorbed methane molecules within Cu-BTC under the bulk phase of low-temperature liquid, related to Figure 2. (Data are represented as the mean value)

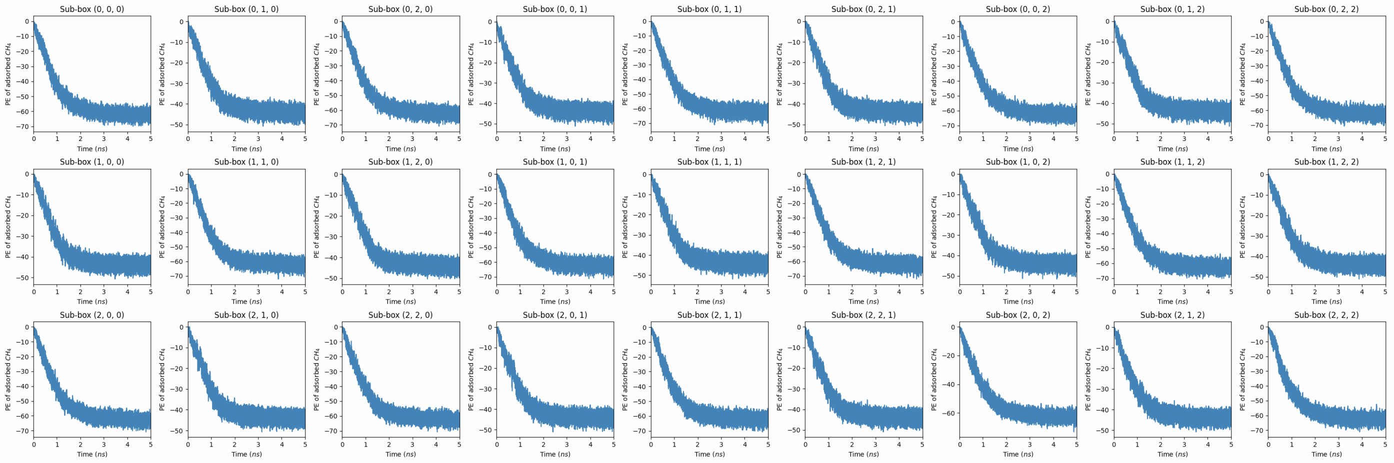

Figure S8. Potential energy of adsorbed methane molecules within Cu-BTC under the bulk phase of low-temperature gas, related to Figure 2. (Data are represented as the mean value)

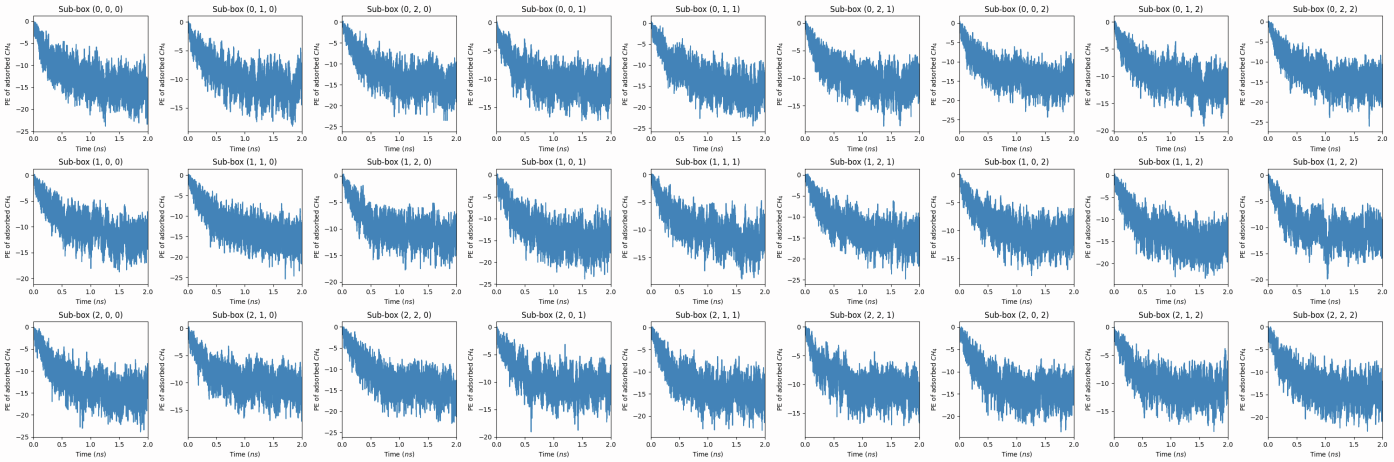

Figure S9. Potential energy of adsorbed methane molecules within Cu-BTC under the bulk phase of room-temperature gas, related to Figure 2. (Data are represented as the mean value)

Figures S7-S9 describe the sum of the potential energy of adsorbed methane molecules in each sub-box according to three bulk phases. Unlike kinetic energy, the exchange of potential energy is more active in high-temperature adsorption, which does not mainly depend on the liquid or gaseous phase.

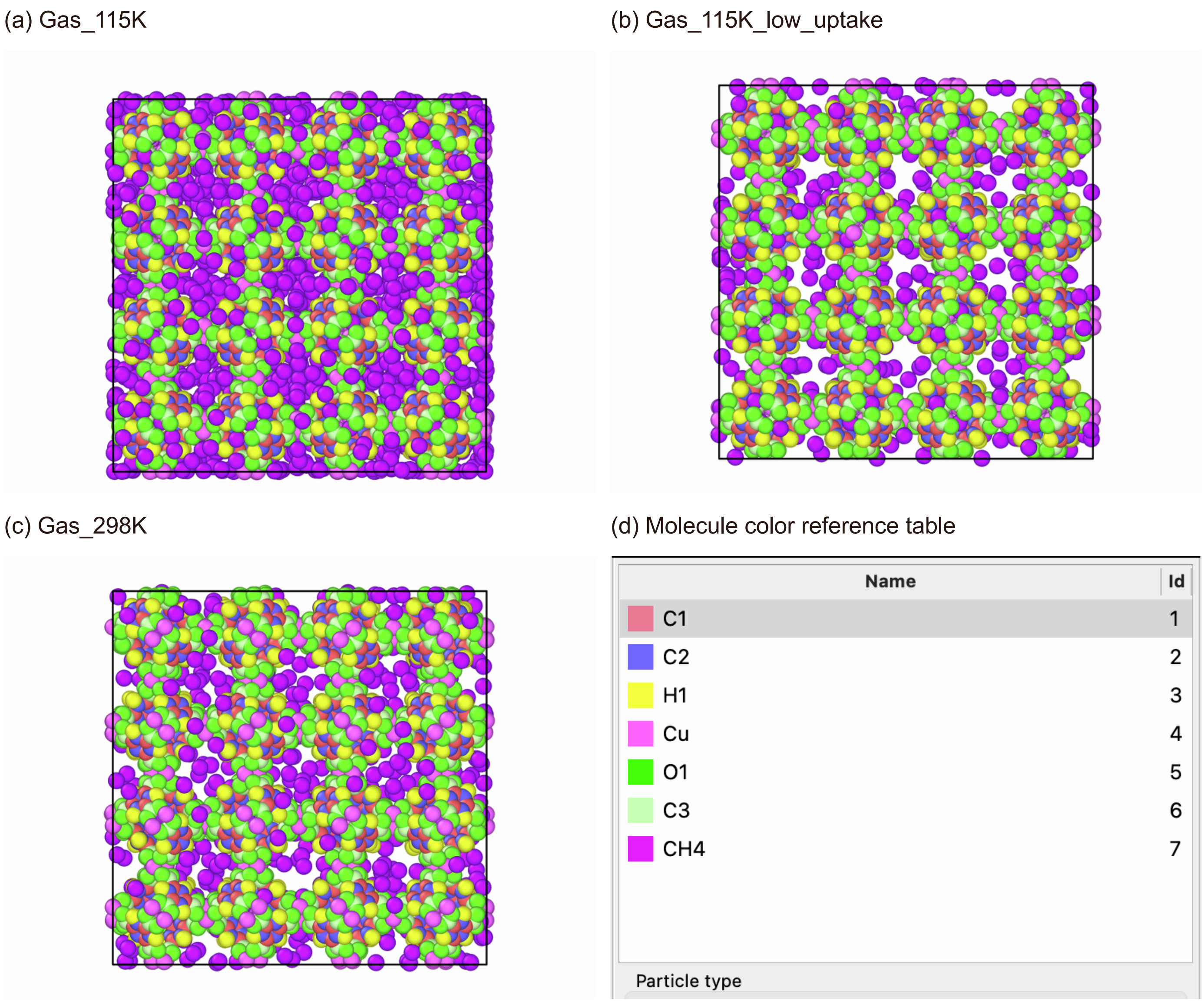

Figure S10. Snapshots of equilibrium adsorption simulation of methane within Cu-BTC under different bulk phases, related to Figure 5.

(a) low-temperature gas, (b) low-temperature gas but low uptake, and (c) room-temperature gas. (d) Molecule color reference table.

Visualized by OVITO.

Notice: (b) and (c) have the same number of methane molecules, but the simulation temperature differs, as mentioned in Figure 5d.

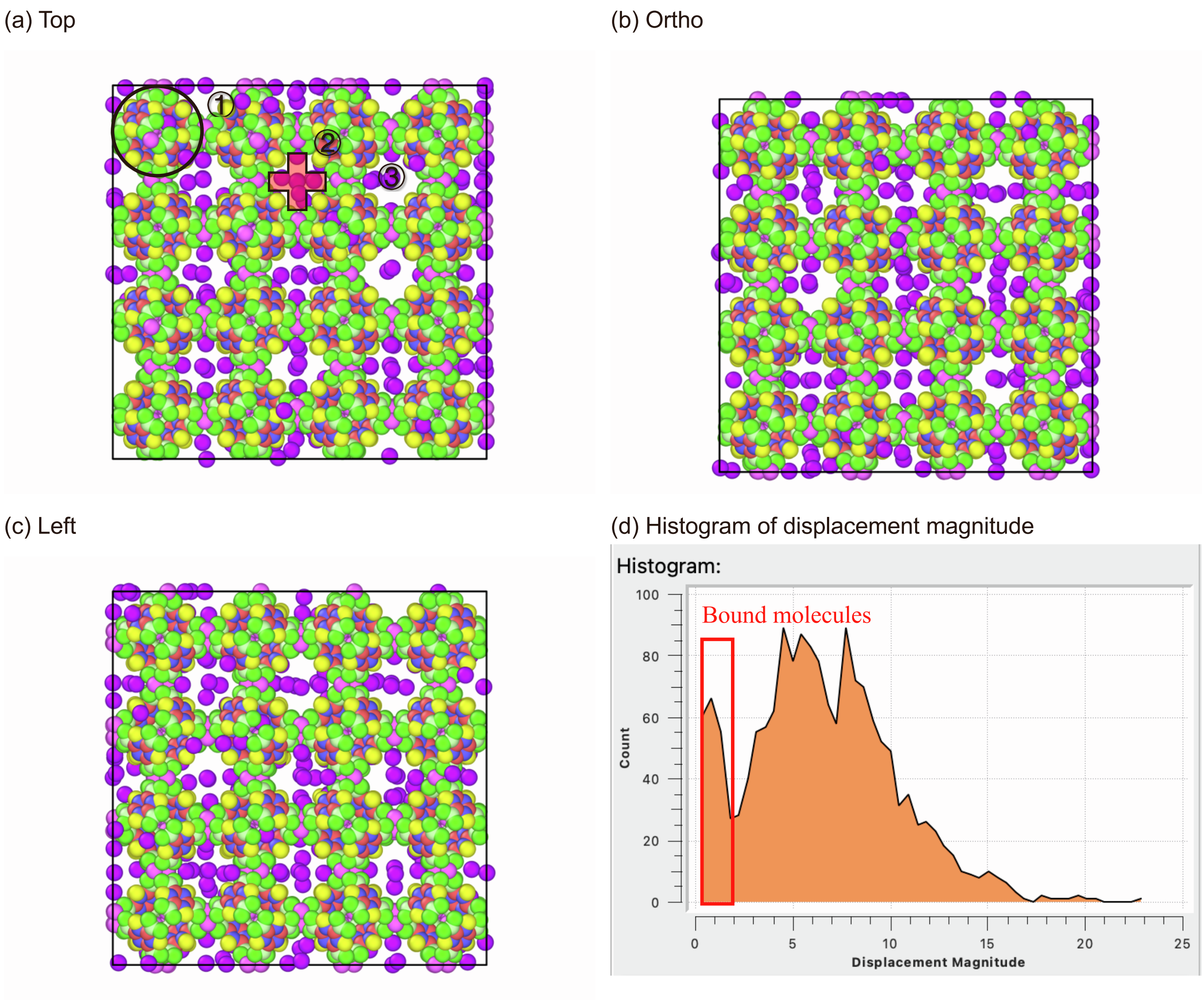

Figure S11. Bound molecules identification under the bulk phase of low-temperature gas, related to Figure 5. (a) (b) (c) Snapshot of bound methane molecules within Cu-BTC under the bulk phase of low-temperature gas. (d) Bound molecules reference for judging with 200 ps intervals.

From Figure S10 (a), take the intervals of 200 ps, the bound molecules distributions are shown in Figure S11 (a) (b) and (c). According to the snapshot, there are three kinds of bound molecules. The first kind is the molecules hiding within the joints. In Cu-BTC, those joints contain narrow space, which allows methane molecules to stay. The second kind is the molecule distributed near the copper atoms (pink color). The third kind is the molecule in the center of each cage. With the second and the third kind molecules, the distribution of them is likewise a “cross” in the cages.

(a) Top

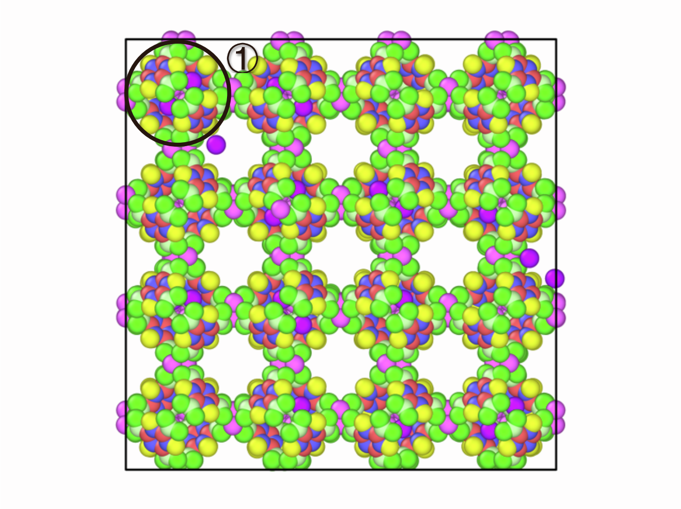

(b) Ortho

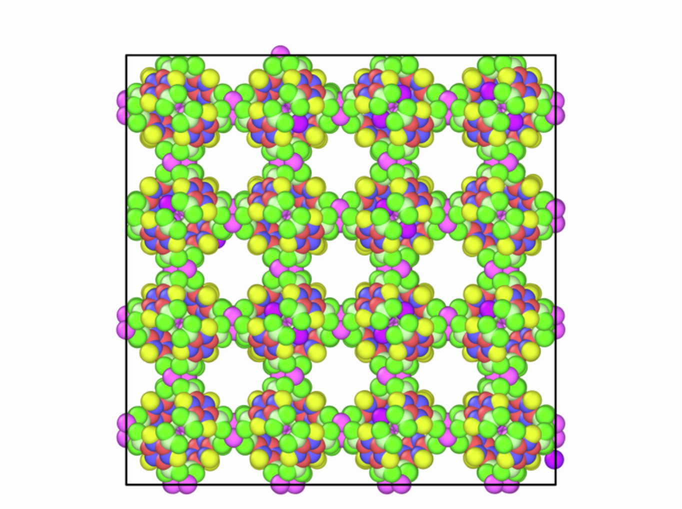

(c) Left

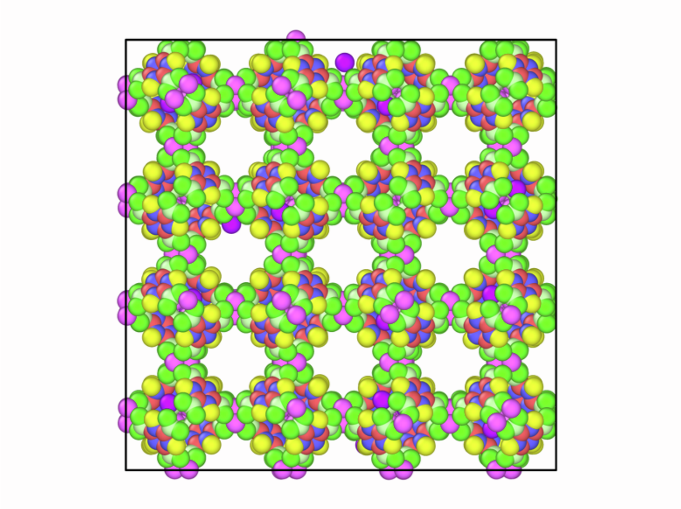

(d) Histogram of displacement magnitude

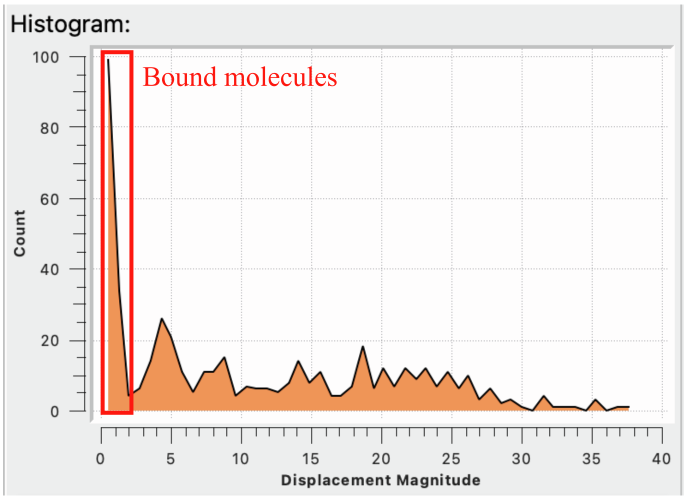

Figure S12. Bound molecules identification under the bulk phase of low-temperature gas with low uptake, related to Figure 5.

(a) (b) (c) Snapshot of bound methane molecules within Cu-BTC under the bulk phase of low-temperature gas but low uptake. (d) Bound molecules reference for judging with 200 ps intervals.

From Figure S10 (b), take the intervals of 200 ps, the bound molecules distributions are shown in Figure S12 (a) (b) and (c). According to the snapshot, excluding the molecules that are not statistically significant, there is only one kind of bound molecule, which is hiding within the joints.

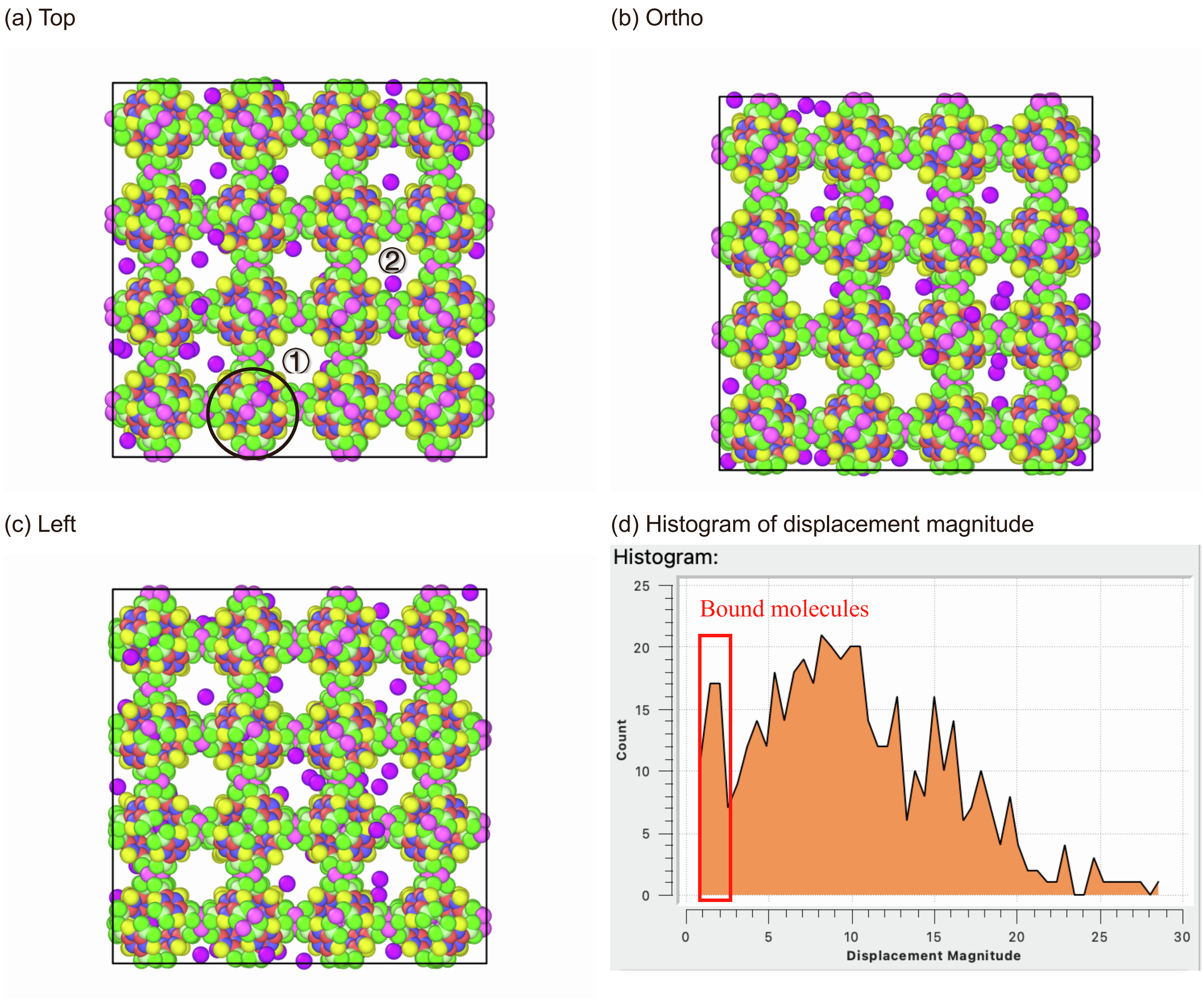

Figure S13. Bound molecules identification under the bulk phase of room-temperature gas, related to Figure 5.
(a) (b) (c) Snapshot of bound methane molecules within Cu-BTC under the bulk phase of room-temperature gas. (d) Bound molecules reference for judging with 10 ps intervals.

From Figure S10 (c), take the intervals of 10 ps, the bound molecules distributions are shown in Figure S13 (a) (b) and (c). According to the snapshot, there are two kinds of bound molecules. The molecules hide within the joints and the molecules near the copper atoms. In equilibrium adsorption simulation, the number of methane molecules is the same as Figure S10 (b), but the bond molecules act in different distributions, indicating that temperature is a significant parameter.

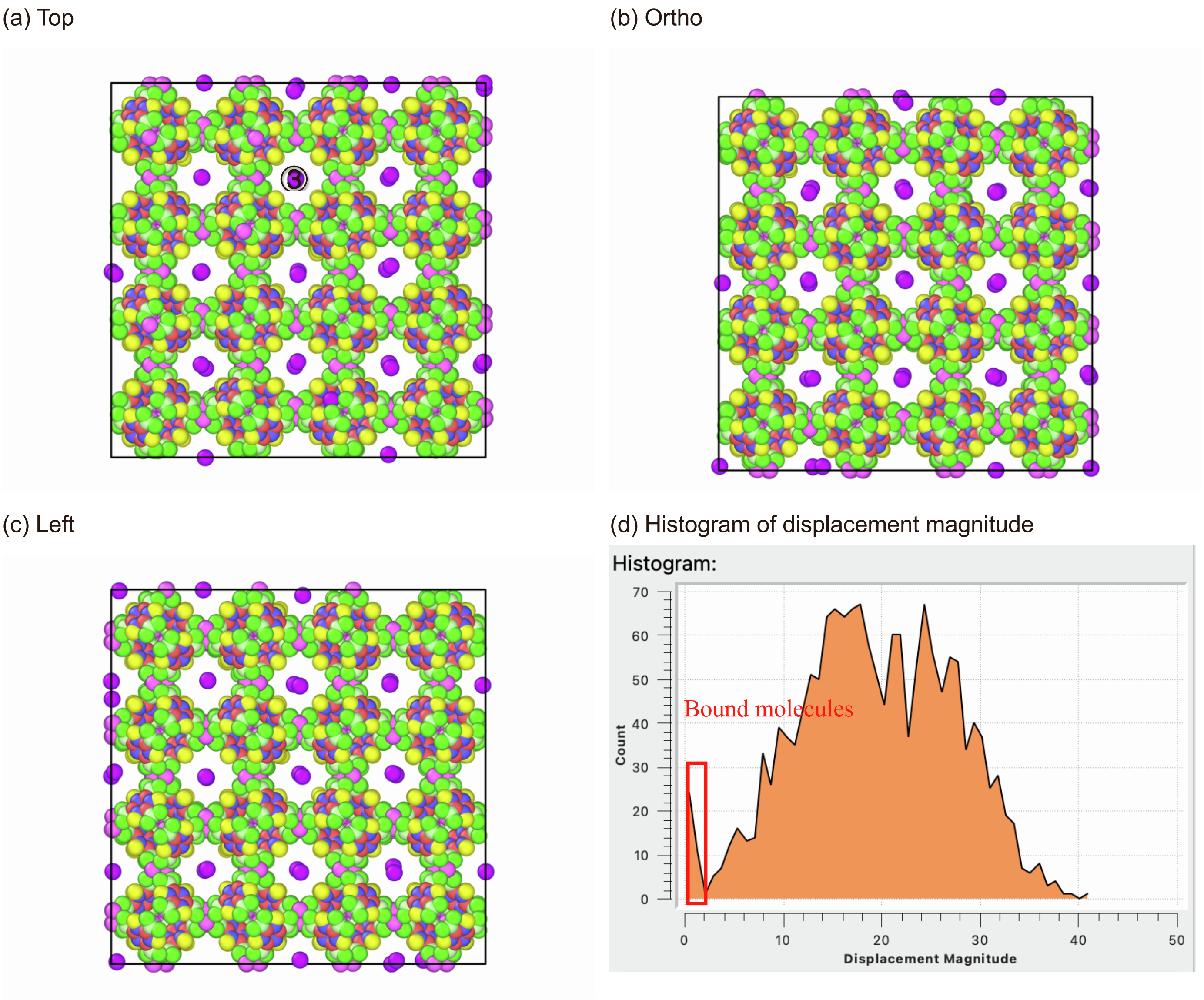

Figure S14. Bound molecules identification under the bulk phase of low-temperature gas with long time interval, related to Figure 5.

(a) (b) (c) Snapshot of bound methane molecules within Cu-BTC under the bulk phase of low-temperature gas with long time interval. (d) Bound molecules reference for judging with 2000 ps intervals.

In Figure S10, the “cross” distribution phenomena are observed. To figure this out, we take the intervals of 2000 ps from Figure S10 (a). According to Figure S14, only one kind of bound molecule is left, the molecule in the center of each cage, which is quite different from the traditional adsorption sites. Therefore, the bound molecule depends not only on the adsorption sites of the adsorbent but also on the adsorbed phase of the adsorbate.

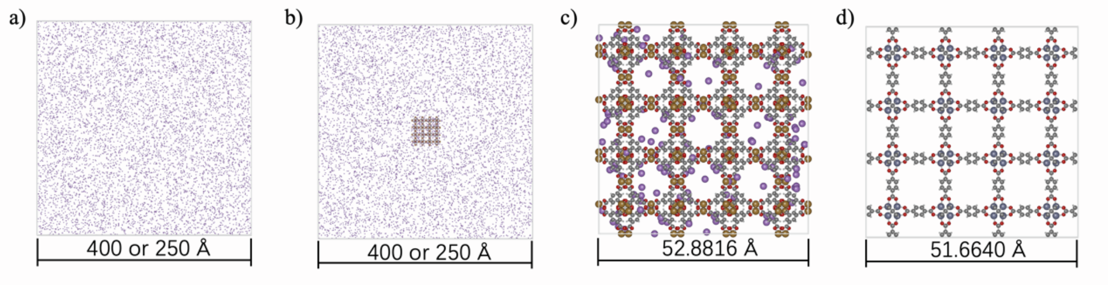

Figure S15. Simulation schematics, related to STAR Methods.
(a) bulk phase creation, (b) adsorption process simulation, and (c) equilibrium adsorption simulation with methane and Cu-BTC. (d) The supercell of MOF-5.

Table S1. LJ interaction of methane and Cu-BTC, related to STAR Methods.

**Atom**	**ε (kcal/mol)**	**σ (Å)**
CH_4-C1	0.1672	3.60
CH_4-C2	0.1672	3.60
CH_4-C3	0.1672	3.60
CH_4-Cu	0.0383	3.42
CH_4-O	0.1678	3.38
CH_4-H	0.0669	3.28
CH_4-CH_4	**0.2941**	**3.72**

Table S2. LJ interaction of methane and MOF-5, related to STAR Methods.

**Atom**	**ε (kcal/mol)**	**σ (Å)**
CH_4-C	0.0951	3.47
CH_4-O_2	0.0957	3.03
CH_4-O_R	0.0957	3.03
CH_4-Zn	0.0550	4.05
CH_4-H	0.0152	2.85
CH_4-CH_4	**0.3150**	**3.72**

Notice: Table S1 and Table S2 have different LJ interactions for CH_4-CH_4, because they are applied in different adsorption pairs and both are verified with experimental adsorption isotherms.
